# A Biosignal-Driven Wearable System Integrating Surface Electromyography and Kinematics for Interpretable Upper-Limb Assessment

**DOI:** 10.3390/bios16100590

**Published:** 2026-10-21

**Authors:** Yu-Chi Wu, Kuo-Yin Lee, Hao-Pu Lin, Chin-Chuan Han, Yuan-Yang Cheng

**Affiliations:** 1 Department of Electrical Engineering, National United University, Miaoli 36003, Taiwan; iopbenson@gmail.com; 2 National Health Research Institute, Miaoli County 35053, Taiwan; haopu@nhri.edu.tw; 3 Department of Computer Science and Information Engineering, National United University, Miaoli 36003, Taiwan; cchan@nuu.edu.tw; 4 Department of Post-Baccalaureate Medicine, Intelligent Long Term Medical Care Research Center, College of Medicine, National Chung Hsing University, Taichung 40705, Taiwan; rifampin@nchu.edu.tw; 5 Department of Physical Medicine and Rehabilitation, Taichung Veterans General Hospital, Taichung 40705, Taiwan

**Keywords:** wearable biosensor, surface electromyography, neuromuscular assessment, multimodal sensing, dynamic time warping (DTW), kinematics, 1D convolutional neural network (1D-CNN), upper-limb rehabilitation

## Abstract

Continuous monitoring of neuromuscular and kinematic behaviors is crucial for effective upper-limb rehabilitation. This study presents a biosignal-driven, wrist-worn multimodal sensing system that combines discrete 12-class functional hand-gesture recognition with an exploratory multimodal physical interpretation of motion deviations. The platform integrates a surface electromyography (sEMG) envelope sensor for macroscopic motor unit recruitment, dual force-sensitive resistors (FSR) for end-effector contact force modulation, and an inertial measurement unit (IMU) for spatial–temporal biokinematics. Data collected from 20 healthy adults (12,000 one-second trials, each containing 100 synchronized time points at 100 Hz) demonstrated that a one-dimensional convolutional neural network (1D-CNN) achieves 81.23% ± 16.74% accuracy under subject-independent leave-one-subject-out (LOSO) cross-validation, capturing natural inter-individual neuromuscular variations. For baseline within-dataset comparison, a class-stratified random split yielded a macro F1-score of 96.59%. To bridge the gap between classification and clinical interpretation, we implemented a multimodal Dynamic Time Warping (DTW) pipeline. This approach successfully identified maximal-difference segments (Hotzones) across the aligned biosignals, enabling a qualitative visualization of candidate multimodal physical deviations (sEMG envelope and IMU/FSR kinematic discrepancies) during gesture execution. By establishing gesture-specific reference distributions from healthy subjects, this cost-effective, low-latency IoT biosensor platform demonstrates preliminary technical feasibility. It lays the groundwork for prospective clinical validation in stroke survivors, aiming to capture compensatory mechanisms and support home-based precision rehabilitation.

## 1. Introduction

Upper-limb and hand impairment after stroke can restrict grasping, finger extension, wrist motion, forearm rotation, and object manipulation. Conventional clinical scales remain essential, but they provide intermittent observations and may not capture the complete time course of muscle activation, movement kinematics, and local pressure changes. Wearable sensing and machine-learning methods can complement clinical observation by recording repeatable time-series data and supporting home-based or remote rehabilitation workflows [1,2,3].

Functional movement execution fundamentally originates from neuromuscular drive, making surface electromyography (sEMG) an indispensable biosignal for capturing motor intent and pathological muscle activation patterns. However, relying solely on electrical biosignals fails to quantify the resultant physical interaction with the environment. Standalone kinematic sensors (IMUs) can track spatial displacement but cannot distinguish genuine neurological recovery from pathological compensatory movements. Integrating mechanosensory contact transducers (FSRs) and inertial kinematics provides the necessary physical context to interpret primary sEMG biosignals. This biosignal-driven multimodal framework establishes a coherent diagnostic link between internal neural drive (sEMG), spatial trajectory execution (IMU), and distal contact-force modulation (FSR) for precision home-based rehabilitation assessment [4,5,6,7].

Previous studies have demonstrated multimodal hand-gesture recognition in both clinical and healthy populations. Song et al. combined EMG, force myography, and inertial sensing in a serious-game system evaluated in people after stroke [4]. Sarwat et al. further addressed post-stroke interparticipant variability and sensor displacement through one-shot transfer learning [5]. In healthy adults, Colli Alfaro and Trejos evaluated user-independent EMG–IMU fusion [7], while Zhang et al. and Rohr et al. examined multimodal fusion across wearing positions and subjects [8,9]. These studies show the value of multimodal biosignal fusion, but they also highlight persistent challenges in cross-user generalization and wearing-condition variability.

Subject-independent validation is particularly important for wearable time-series models. Gholamiangonabadi et al. demonstrated that model performance can decrease substantially when evaluation changes from conventional cross-validation to leave-one-subject-out validation [10]. Accordingly, within-dataset random-split performance and LOSO performance should be reported separately because they answer different generalization questions.

Gesture classification alone does not explain how an execution differs from a reference movement. DTW-based approaches have been used to evaluate upper-limb task quality, compare rehabilitation gestures, align sequences with different execution speeds, and construct representative time-series templates [11,12,13,14,15]. FastDTW provides an approximate linear-time alignment method suitable for interactive analysis [16]. The alignment path can also support temporal localization and channel-level interpretation of movement differences. In an unconstrained telerehabilitation setting, automated gesture recognition serves three operational purposes: (1) verifying compliance by confirming that the patient performs the prescribed exercise rather than an unintended movement; (2) automating repetition counting; and (3) routing the incoming trial to its gesture-specific normative template for dynamic time warping (DTW) comparison without requiring manual logging. The 12 target classes were selected from the functional domains of the Fugl-Meyer Assessment (FMA-UE) and Action Research Arm Test (ARAT), encompassing gross finger flexion/extension (MF, ME), grasp synergies (HG, CG, SG), fine precision manipulation (TA, O), and wrist/forearm stabilization (WE, WF, FP, FS), alongside a baseline resting state (NM).

The present study developed a wrist-worn system integrating one sEMG channel, two mechanosensory contact-force transducer channels, and one IMU. The objectives were to: (1) construct a synchronized multimodal gesture dataset; (2) compare CNN and MLP classifiers across nine sensor configurations; (3) evaluate sample-wise performance, paired model differences, LOSO generalization, and re-donning stability; and (4) develop an interpretable DTW workflow reporting normalized distance, channel contribution, and maximal-difference segments. Because all participants were healthy adults, the work is strictly positioned as an engineering proof-of-concept and technical feasibility study rather than a validated clinical diagnostic assessment. Prospective clinical validation in patient populations will be required to establish diagnostic efficacy.

## 2. Materials and Methods

### 2.1. System Overview

**Figure 1:**
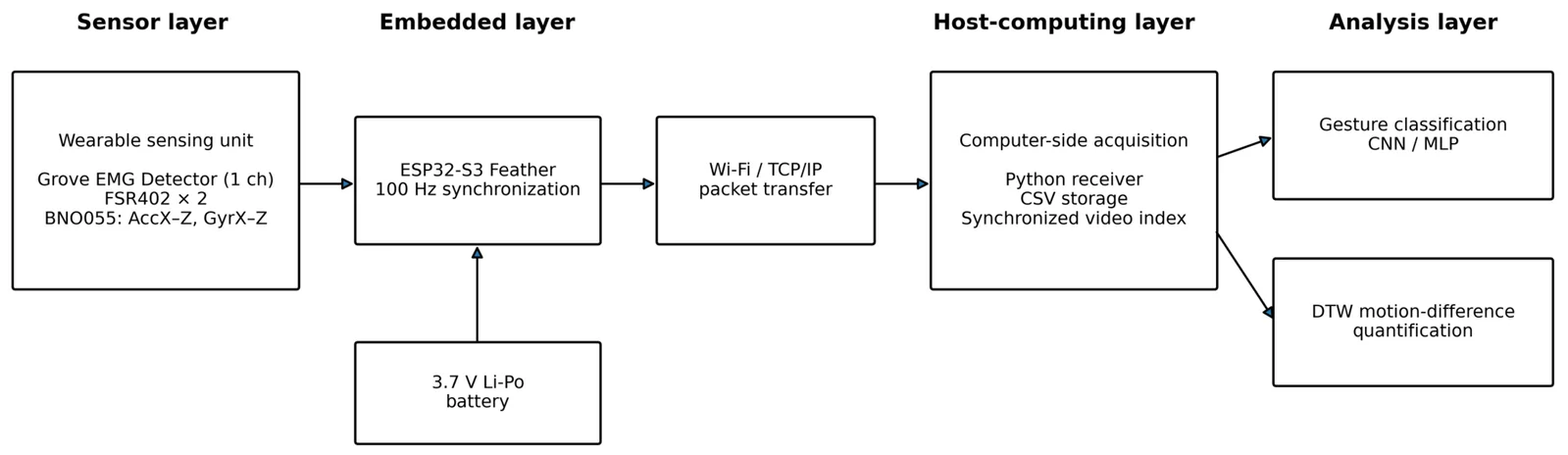
System architecture of the wrist-worn multimodal sensing and analysis platform.

**Figure 2:**
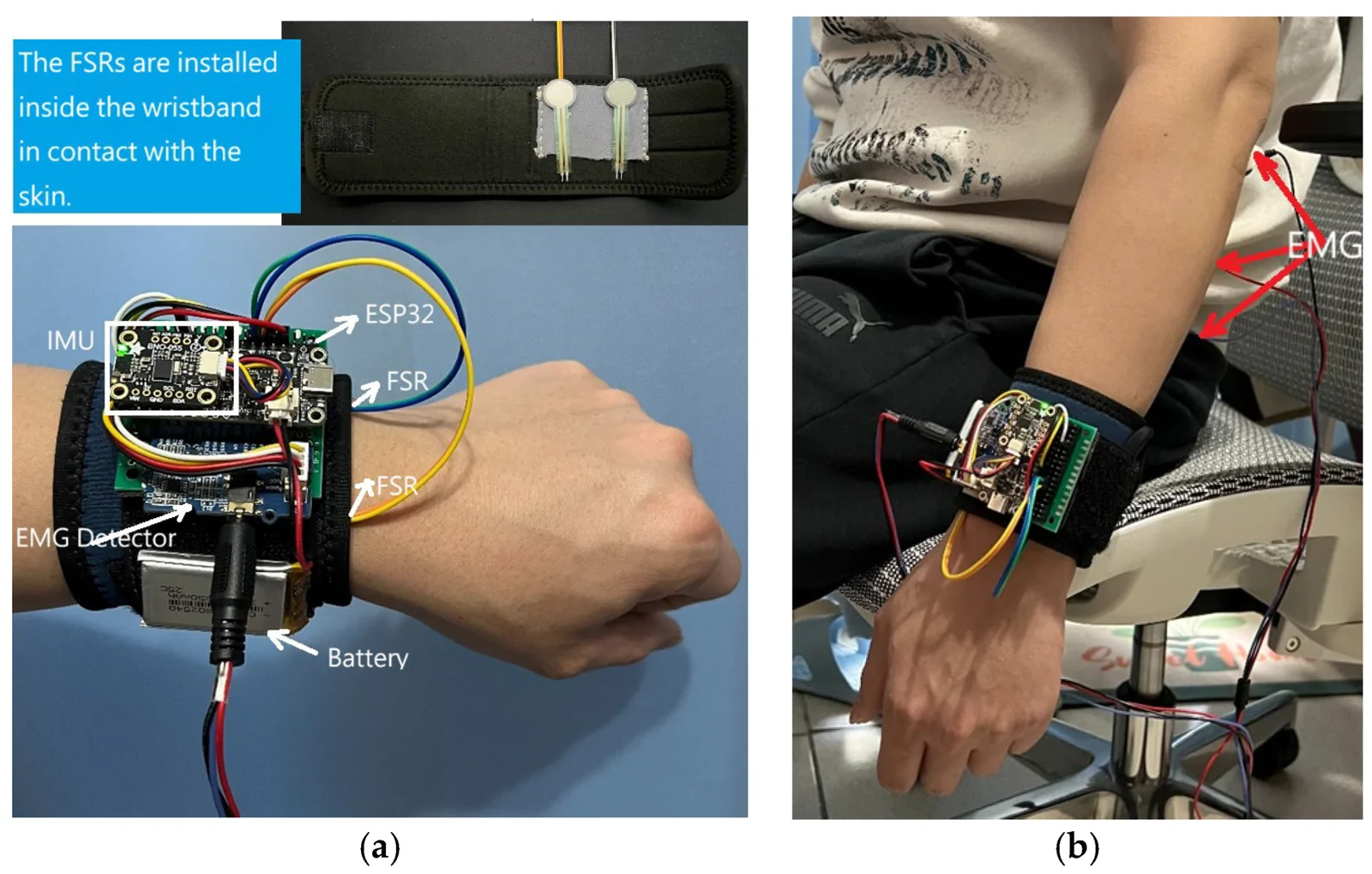
(**a**) Prototype wrist-worn device; (**b**) device worn on the participant’s left wrist.

The wearable platform comprised a Grove EMG Detector [17], two FSR402 sensors [18], a BNO055 inertial sensor [19], and an Adafruit ESP32-S3 Feather microcontroller based on the ESP32-S3 platform [20]. A 3.7 V lithium-polymer battery enabled untethered operation. The microcontroller sampled and synchronized the sensor channels and transmitted packets to a computer over Wi-Fi using TCP/IP. Video was recorded separately for DTW visualization and was not used as an input to the CNN or MLP classifiers. The overall system architecture is illustrated in Figure 1, and the prototype device and its wearing configuration are shown in Figure 2. The sEMG electrodes were positioned over the forearm flexor digitorum superficialis muscle belly to capture compound finger and wrist flexion drives. The dual FSR transducers were installed on the interior wristband lining at radial and ulnar contact zones to measure localized interface pressure variations induced by tendon expansion and muscular deformation, rather than direct fingertip-object contact forces.

The pipeline operates as a near-real-time trial-by-trial evaluation system. System latency includes the fixed 1.0 s buffered motion window (100 samples) and Wi-Fi TCP/IP socket transmission (12.4 ± 2.8 ms). Real-time inference latency benchmarking was performed on a consumer-grade host laptop (Intel Core i7-12700H) without reliance on GPU acceleration. The evaluation confirmed that the host-side 1D-CNN forward inference requires only 2.14 ± 0.31 ms, verifying that the proposed lightweight architecture (213,964 parameters) achieves sub-3-ms inference on standard CPU hardware alone. This aligns perfectly with the system’s goal of low-cost, GPU-independent deployment for home-based telerehabilitation. Combined with the FastDTW alignment (18.6 ± 3.2 ms), the pipeline yields a computational processing delay of ≈33 ms post-movement termination. When incorporating the fixed 1.0 s buffered data acquisition window, this results in a total system latency of ≈1033 ms from initial movement onset. The pipeline operates with a packet loss rate of <0.05% over 12,000 trials.

### 2.2. Sensors and Data Acquisition

The classification input comprises nine synchronized channels: one sEMG envelope channel, two mechanosensory contact-force transducers (FSRs), three-axis acceleration, and three-axis angular velocity, sampled continuously at a unified rate of 100 Hz. To construct a standardized, well-aligned gesture dataset across 20 participants, a structured motion-segmentation protocol was established. Each active gesture trial was initiated from a standardized relaxed arm-down baseline posture (arm naturally aligned with the torso, elbow extended, and hand relaxed).

For active gesture classes, physical motion onset was detected using an acceleration magnitude threshold:

(1) $$\sum_{i\in \left\{x,y,z\right\}}\left|a_{i}\right|\geq \theta_{\mathrm{acc}}$$

where $\theta_{\mathrm{acc}}=18.0$ (in software acceleration units). Summing the absolute values across all three orthogonal axes prevents directional vector cancellation and robustly differentiates intentional limb transit from minor resting tremor. Upon crossing this threshold, the ESP32-S3 microcontroller synchronously buffered the subsequent 100 consecutive multimodal samples, capturing a fixed 1-s dynamic window (100 × 9 matrix).

Conversely, the baseline ‘No Motion’ (NM) class represents steady-state resting neuromuscular tone without gross limb displacement. Because baseline resting tone cannot and should not cross kinetic thresholds, the host computer issued a dedicated DATASERVER_NM software command. This triggered a condition-bypass routine in the firmware, directly logging an un-triggered 1-s stationary window to preserve baseline muscle tone and ambient contact pressure without temporal truncation.

*Rationale for Protocol-Specific Kinematic Triggering:*

In this laboratory benchmark study, all 11 active gesture protocols inherently involved gross wrist, forearm, or hand transit initiated from the resting arm-down posture, making the kinematic threshold exceptionally consistent and impervious to baseline sEMG electrode impedance fluctuations. Nonetheless, we recognize that in unconstrained, continuous ambulatory monitoring, pure isometric contractions (e.g., sustained static grip or power grasp executed without arm displacement) exhibit negligible spatial acceleration and could evade an acceleration-only trigger. To address continuous real-world deployments, the embedded firmware architecture is inherently designed to execute a generalized hybrid onset trigger:

(2) $$T_{\mathrm{atv}}=\left(\sum_{i\in \left\{x,y,z\right\}}\left|a_{i}\right|\geq \theta_{\mathrm{acc}}\right)|\vee |\left({\mathrm{EMG}}_{\mathrm{Env}}\geq \theta_{\mathrm{EMG}}\right)\;$$

where ∨ denotes the logical OR operator and $\theta_{\mathrm{EMG}}$ represents an adaptive threshold above the baseline electrical noise floor. For the present offline benchmarking and DTW reference construction, the kinematic threshold provided unambiguous, deterministic trial alignment across all active gestures. While the current standardized laboratory protocol was completed within the fixed 1.0 s acquisition window across healthy subjects, prospective deployment for unconstrained continuous movements will utilize an adaptive sliding buffer that logs samples until sub-threshold kinetic tone is restored, with movement duration variations decoupled and normalized via the reported Kinematic Duration Ratio ($T_{ratio}$).

**Figure 3:**
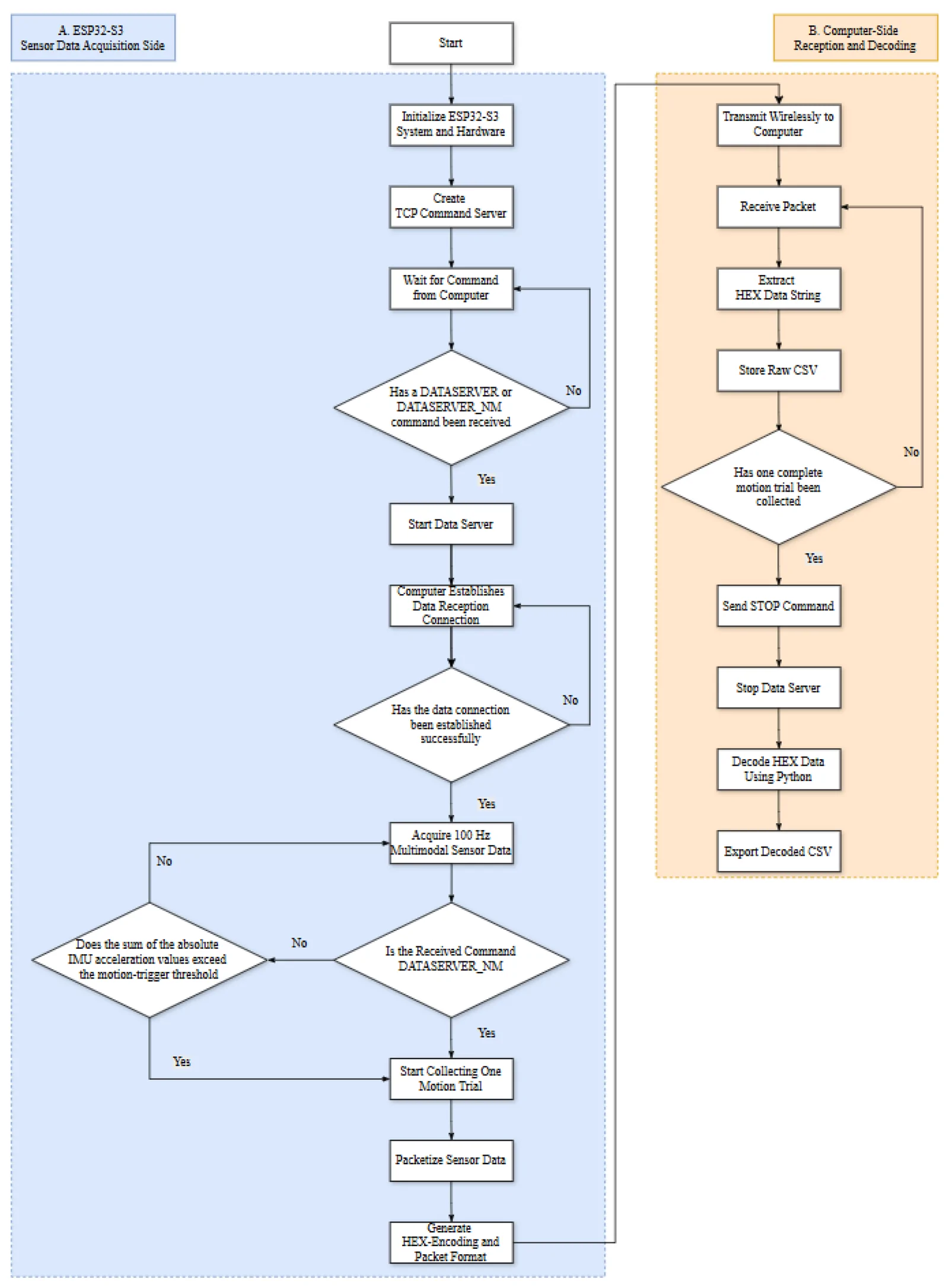
Wireless data transmission, reception, and storage workflow.

The end-to-end wireless acquisition, transmission, reception, and storage pipeline is summarized in Figure 3. On the wearable side, the ESP32-S3 initializes the sensing system, waits for a command from the computer, and records a one-second motion segment when the acceleration threshold is exceeded or when a no-motion command is issued. On the computer side, incoming packets are received, stored, decoded, and exported as CSV data for subsequent analysis. The sensing channels and their primary analytical roles are summarized in Table 1.

#### Biosensing Transduction Principles and Multimodal Signal Fidelity

From a biosensor transduction perspective, conventional high-density electromyography systems require multi-kilohertz sampling rates, imposing excessive data payloads, severe power dissipation, and cumbersome skin-preparation protocols that hinder continuous home monitoring. In the context of macroscopic gesture discrimination and motor deficit localization, the primary physiological information resides within the low-frequency modulation of motor unit recruitment envelopes—specifically, the temporal onset, burst duration, and amplitude dynamics of the compound muscle action potential. The hardware-level rectification and active low-pass filtering embedded in the analog sEMG sensor directly extract this macroscopic activation envelope prior to digitization. This analog-domain feature extraction reduces the bandwidth demand, enabling the onboard ESP32-S3 microcontroller to achieve microsecond-level synchronization across the sEMG envelope, mechanosensory contact-force transducers (FSRs), and 6-DOF biokinematic streams (IMU) at a unified 100 Hz rate with ultra-low packet-transmission latency. Furthermore, the dual FSR transducers serve as physical mechanosensory interfaces, capturing distal end-effector contact timing and relative grip-force modulation without requiring rigid mechanical dynamometry. This integrated biosensing architecture optimizes biological signal fidelity against wearable ergonomic and energy constraints for scalable telerehabilitation deployment.

### 2.3. Participants and Gesture Protocol

Twenty healthy adults participated. The mean age was 27.2 ± 4.27 years (range, 20–35); 18 participants were male and 2 were female. Eighteen were right-hand dominant and two were left-hand dominant. Participants reported no hand-related medical history. All recordings were performed with the device on the left hand between January and May 2026.

**Figure 4:**
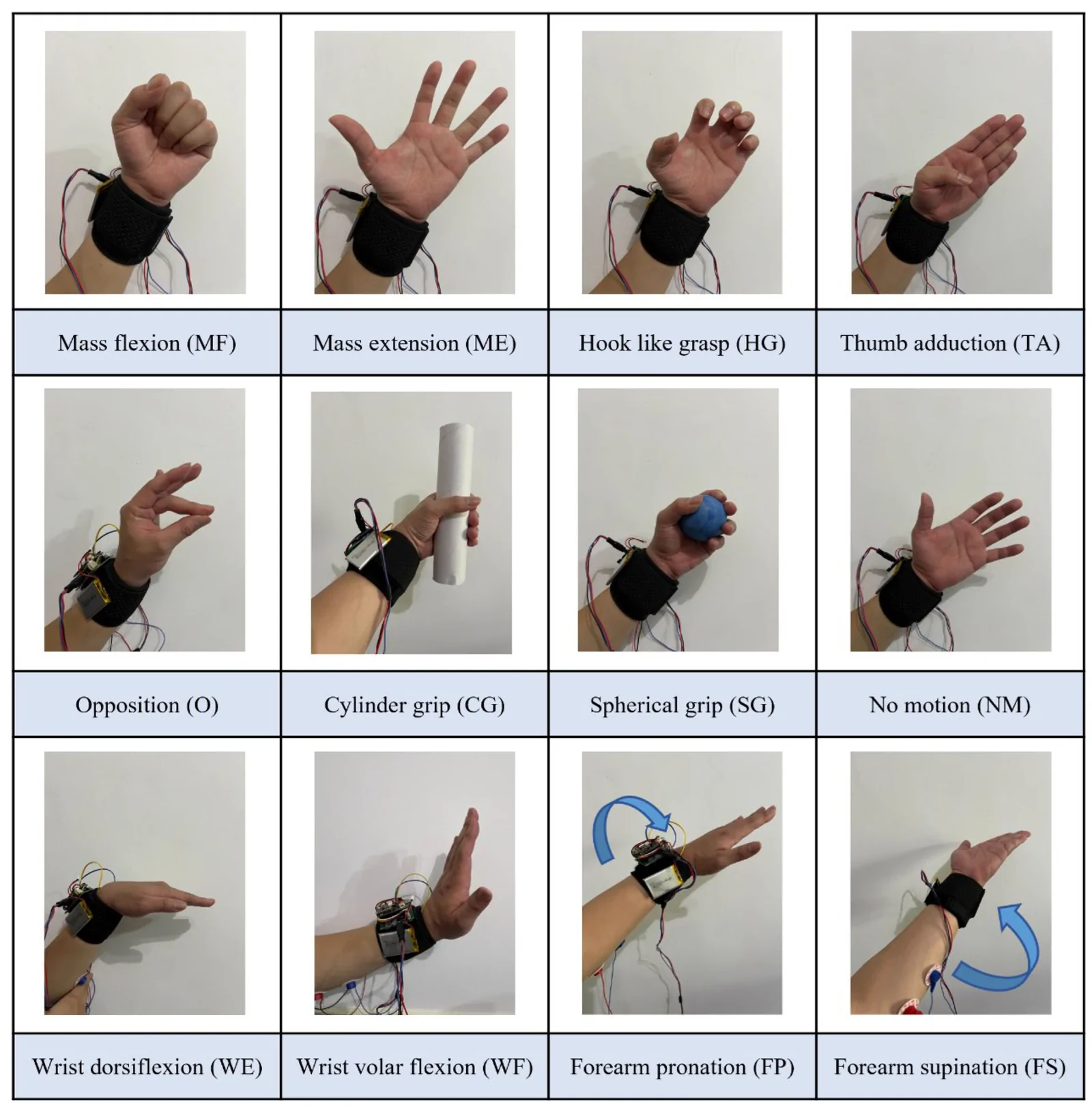
The 12 hand, wrist, and forearm gesture classes used for dataset construction.

The protocol included 12 classes: mass flexion (MF), mass extension (ME), hook-like grasp (HG), thumb adduction (TA), opposition (O), cylinder grip (CG), spherical grip (SG), no motion (NM), wrist extension/dorsiflexion (WE), wrist flexion/volar flexion (WF), forearm pronation (FP), and forearm supination (FS). Each participant performed 50 repetitions per class, producing 600 samples per participant and 12,000 samples overall. All active gestures began from a standardized relaxed arm-down posture and were completed in approximately one second. The 12 gesture classes are illustrated in Figure 4, and the participant and dataset characteristics are summarized in Table 2.

### 2.4. Preprocessing and Sensor Configurations

Each complete multimodal sample was represented as a 100 × 9 matrix. Nine sensor configurations were evaluated: complete multimodal input (C = 9); EMG + IMU (C = 7); FSR + IMU (C = 8); EMG + FSR (C = 3); IMU only (C = 6); EMG only (C = 1); two mechanosensory contact-force transducers (both FSR channels) (C = 2); single mechanosensory contact-force transducer (FSR_0 only) (C = 1); and another single mechanosensory contact-force transducer (FSR_1 only) (C = 1). CNN inputs retained the 100 × C time-by-channel form, whereas MLP inputs were flattened into 100C-dimensional vectors.

The original sensor measurements were normalized using predefined hardware-specific scaling factors (e.g., EMG and FSR values were divided by their 12-bit ADC maximum resolution of 4096) prior to dataset partitioning. Notably, traditional physiological normalization methods, such as Maximum Voluntary Contraction (MVC) calibration for sEMG, were intentionally omitted. This fixed-factor engineering normalization was selected to evaluate the system’s out-of-the-box feasibility for zero-calibration home-use scenarios. In such remote rehabilitation contexts, requiring stroke patients to perform maximal or controlled sub-maximal contractions prior to daily use is often physically impractical, potentially unsafe, and clinically contraindicated.

### 2.5. Classification Models

For the complete multimodal configuration, the MLP flattened each 100 × 9 sample into a 900-dimensional input vector. The six hidden dense layers contained 1024, 512, 512, 64, 64, and 32 units with rectified linear unit activation. Dropout rates of 0.2 and 0.4 were applied after the first 512-unit layer and the first 64-unit layer, respectively. A 12-unit softmax layer produced the class probabilities.

**Figure 5:**
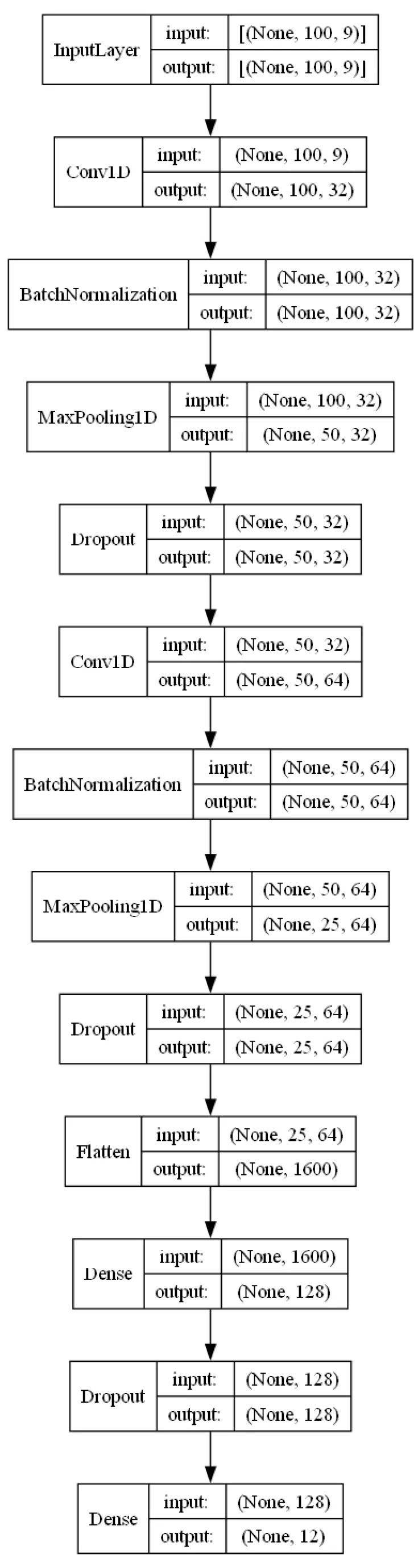
Detailed layer-by-layer architecture and tensor dimensions of the proposed 1D-CNN used for gesture recognition. The input layer shape (100, 9) illustrates the complete multimodal configuration (C=9 channels).

The one-dimensional CNN directly accepted the 100 × C time-series matrix. The first convolutional block used 32 filters with a kernel size of 3, same padding, rectified linear unit activation, batch normalization, max pooling with a pool size of 2, and dropout at a rate of 0.1, yielding a $\left(50,32\right)$ feature map. The second block used 64 filters with the same kernel size and padding, followed by batch normalization, max pooling, and dropout at a rate of 0.2, yielding a $\left(25,64\right)$ feature map. The resulting feature map was flattened into a 1600-dimensional vector and passed through a 128-unit dense layer with rectified linear unit activation and dropout at a rate of 0.3, followed by a 12-unit softmax output layer. One-dimensional CNNs are well suited to multichannel wearable time series and have also been applied to rehabilitation exercise assessment [12]. The complete layer-by-layer 1D-CNN architecture and tensor transformations are detailed in Figure 5.

Software models were implemented in Python 3.10.19 using TensorFlow/Keras 2.12.0, NumPy 1.23.5, Pandas 2.3.3, SciPy 1.15.3, Scikit-learn 1.7.2, Matplotlib 3.10.7, and FastDTW 0.3.4. Both models were compiled using categorical cross-entropy loss and the Adam optimizer with default parameters ($\beta_{1}=0.9$, $\beta_{2}=0.999$, $\epsilon =1\times 10^{-7}$, initial learning rate $\eta =1\times 10^{-3}$). Training was conducted with a batch size of 16 for a maximum of 70 epochs. A ReduceLROnPlateau callback monitored validation loss, reducing the learning rate by a factor of 0.5 after eight epochs without improvement, down to a minimum learning rate of $1\times 10^{-6}$. To ensure analytical reproducibility, a global random seed of 1337 was applied to NumPy, TensorFlow, and dataset partitioning. To ensure rigorous and comparable benchmarking across all sensor configurations (Section 3.1), the offline 10-seed model training and batch evaluation were performed on a computing environment equipped with an NVIDIA A100 GPU (40 GB HBM2e), CUDA 11.8, and cuDNN 8.6, ensuring that the reported training-time measurements are directly and internally comparable across all nine sensor configurations and both model architectures. On the wearable embedded side, firmware was developed using ESP-IDF v4.4.7/Arduino core v2.0.17 on the ESP32-S3 microcontroller (240 MHz clock frequency). The internal 12-bit ADC was configured with 11 dB attenuation (0–3.1 V dynamic range), and the I^2^C bus clock rate was set to 400 kHz.

### 2.6. Evaluation and Statistical Analysis

The normalized samples were divided using a class-stratified sample-wise random split. First, 20% of the samples were assigned to the test set. The remaining 80% were then divided into training and validation subsets, resulting in final proportions of 60%, 20%, and 20%, respectively. Accuracy, weighted precision, weighted recall, weighted F1-score, macro F1-score, and confusion matrices were calculated. Macro F1-score was obtained by calculating the F1-score independently for each of the 12 gesture classes and taking their unweighted arithmetic mean.

CNN and MLP predictions on the same 2400-sample test set were compared using McNemar’s test with continuity correction. To evaluate generalization to unseen participants, LOSO cross-validation was performed in 20 folds. In each fold, all 600 samples from one participant were held out for testing, while data from the other 19 participants were used for model development. Ten percent of the development data was assigned to an internal validation subset. Each LOSO model was trained for a maximum of 30 epochs with a batch size of 16; early stopping monitored validation loss with a patience of five epochs and restored the best weights before evaluation. LOSO results are reported as mean accuracy ± standard deviation across the 20 held-out participants.

The sample-wise random split and LOSO evaluation answer different questions. Because samples from the same participant may occur in the training and test subsets of the random split, that analysis estimates within-dataset recognition performance. LOSO provides the more appropriate estimate of subject-independent generalization to an unseen healthy participant [11].

### 2.7. Preliminary Single-Subject Re-Donning Evaluation

One participant from the original dataset removed and re-donned the system after model training and performed 50 repetitions of each class (600 predictions). This experiment assessed same-person sensitivity to reinstallation, not population-level cross-session reliability.

### 2.8. DTW-Based Motion-Difference Quantification

DTW analysis was conducted on five derived channels: acceleration magnitude (ACC_Mag), angular-velocity magnitude (GYR_Mag), an absolute-value moving-average macroscopic neuromuscular activation envelope (EMG_Env), FSR_0, and FSR_1. To prevent amplitude differences from being eliminated by independent trial normalization, both the reference and evaluated sequences were standardized identically using the baseline mean ($\mu_{ref}$) and standard deviation ($\sigma_{ref}$) derived strictly from the gesture-specific healthy reference template ($z=\left(x-\mu_{\mathrm{ref}}\right)/\sigma_{\mathrm{ref}}$). Because this identical transformation retains absolute amplitude attenuation and baseline shifts, the resulting normalized distance evaluates both waveform morphology and relative amplitude discrepancy concurrently, and should not be interpreted as an isolated measure of waveform shape. FastDTW with Euclidean distance and radius r = 1 was applied with equal channel weights [16].

Furthermore, to prevent DTW time-warping from masking temporal bradykinesia, the revised framework reports decoupled evaluation metrics alongside waveform alignment:

(1) **Shape and Amplitude Dissimilarity**, represented by the path-normalized FastDTW distance ($D_{\mathrm{norm}}=D_{\mathrm{DTW}}/L,$ where L is the warping path length);
(2) **Kinematic Duration Ratio** ($T_{\mathrm{ratio}}=T_{\mathrm{test}}/T_{\mathrm{ref}}$) to quantify movement bradykinesia;
(3) **Peak Amplitude Modulation **(${\mathrm{Amp}}_{\mathrm{ratio}}=\max\left(x_{\mathrm{test}}\right)/\max\left(x_{\mathrm{ref}}\right)$).

To account for the fixed one-second (100-sample) acquisition window, the actual movement duration used for $T_{ratio}$ was dynamically extracted by determining the temporal bounds between the initial kinetic trigger crossing ($\sum \left|a_{i}\right|\;\geq 18.0$) and the subsequent return to a stable sub-threshold acceleration baseline. Along the warping path $P={\left\{\left(p_{m},q_{m}\right)\right\}}_{m=1}^{L}$, the relative contribution $C_{k}$ of channel $k\;\in \left\{1,\;\ldots ,\;K\right\}$ (where K = 5) to the total trajectory dissimilarity is computed as:

(3) $$C_{k}=\frac{\sum_{m=1}^{L}{\left(x_{k}\left(p_{m}\right)-y_{k}\left(q_{m}\right)\right)}^{2}}{\sum_{j=1}^{K}\sum_{m=1}^{L}{\left(x_{j}\left(p_{m}\right)-y_{j}\left(q_{m}\right)\right)}^{2}}\times 100\%\;$$

Furthermore, the maximal-difference segment (Hotzone) is identified by evaluating the instantaneous local squared Euclidean distance across all channels along the alignment path:

(4) $$e\left(m\right)=\overset{K}{\underset{k=1}{\sum }}{\left(x_{k}\left(p_{m}\right)-y_{k}\left(q_{m}\right)\right)}^{2}$$

A Hotzone is defined as the contiguous temporal window $\left[m_{\mathrm{start}},m_{\mathrm{end}}\right]$ where $e\left(m\right)\geq \mu_{e}+2\sigma_{e}$ persists for an elapsed duration of at least 100 ms based on original sequence timestamps ($t_{end}-t_{start}\geq 100\;\mathrm{ms}$), preventing warping-path index artifacts from repeated alignment samples. $\mu_{e}$ and $\sigma_{e}$ denote the mean and standard deviation of local alignment errors across the trial. Sensor samples were linked to Video_Frame_ID values recorded during acquisition, allowing the aligned waveforms to be mapped back to the original video frames [14,15].

Gesture-specific healthy-adult reference distributions were constructed using a repeated random subject-level holdout validation protocol to ensure independent evaluation and mitigate single-split bias. In each iteration, 5 randomly selected subjects were held out as an independent test cohort. The remaining 15 subjects were utilized to establish the normative template and distribution parameters. This randomized partitioning was repeated across 20 independent iterations to ensure comprehensive evaluation. Specifically, an ensemble average waveform was first computed from the 750 training trials (50 subjects × 50 trials). The single individual trial with the minimum Euclidean distance to this ensemble average was then selected as the representative medoid template. The training trials were subsequently compared against this template via FastDTW to estimate the baseline parameters (mean, standard deviation, mean + 2SD, and 95th percentile). This template-selection approach differs from DTW barycenter averaging, which synthesizes a centroid sequence from multiple trials [21].

**Figure 6:**
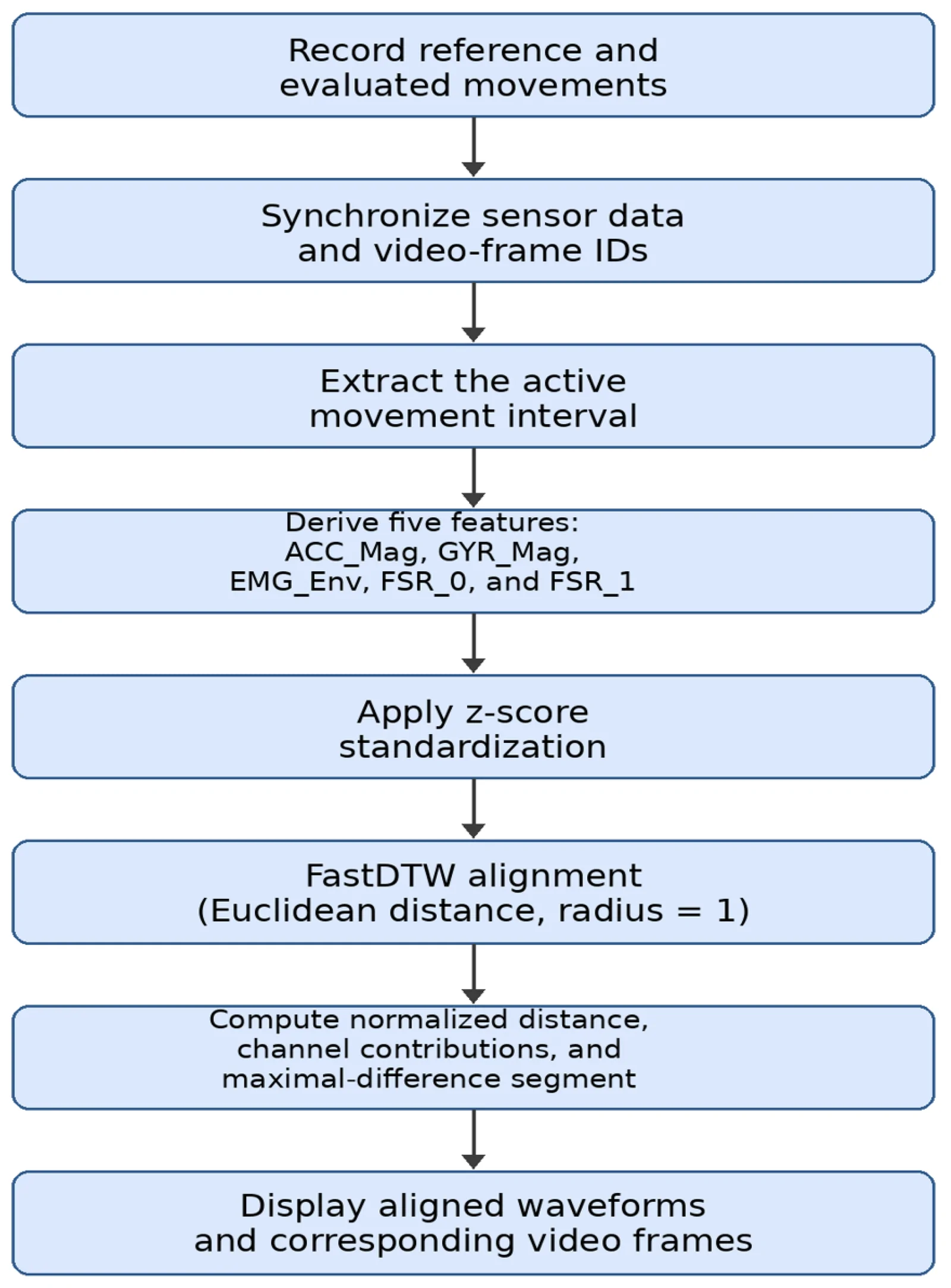
DTW-based motion-difference quantification and synchronized visualization workflow.

To demonstrate the multimodal visualization capabilities of the system’s graphical user interface, preliminary qualitative tests were conducted involving simulated altered movements (e.g., bradykinetic, hypometric). It is crucial to note that these specific trials serve purely as a visual demonstration of the DTW Hotzone layer. They do not represent a quantitative clinical validation, nor are they clinical simulations of stroke-induced neurological pathologies. The complete DTW-based multimodal comparison workflow is summarized in Figure 6.

## 3. Results

### 3.1. Random-Split Classification Performance

On the 2400-sample random test set, the complete multimodal 1D-CNN achieved 96.58% accuracy and a 96.59% macro F1-score, outperforming the baseline MLP (94.00% accuracy, 94.01% macro F1-score). More importantly than raw accuracy, the 1D-CNN architecture inherently leverages localized receptive fields to capture transient physiological phenomena—such as the rapid onset of muscle activation (sEMG envelope peaks) and subsequent physical impact (FSR pressure spikes)—which are critical for neuromuscular assessment.

**Figure 7:**
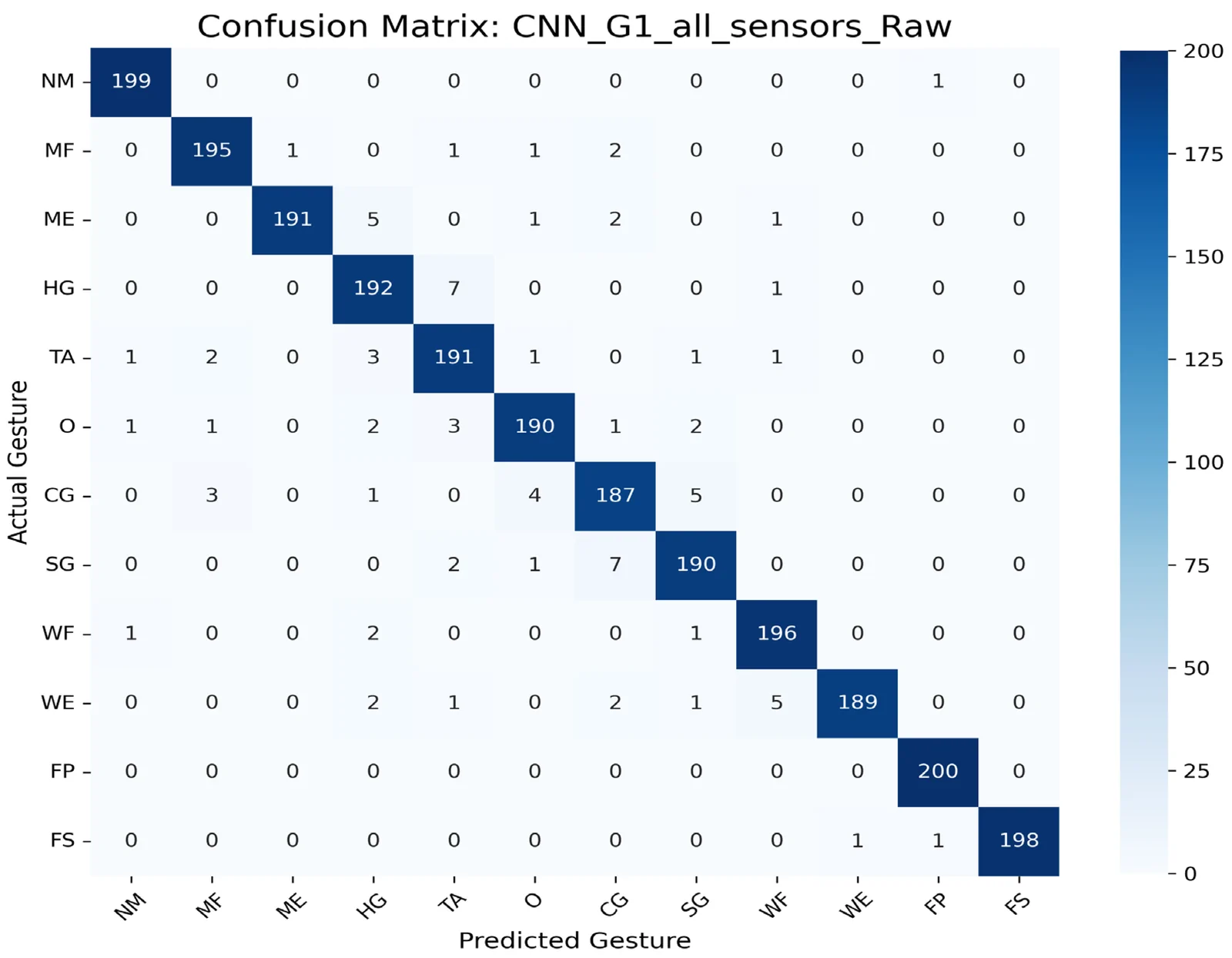
Confusion matrix of the full multimodal CNN on the random test split.

Unlike the MLP, which flattens time series into fixed positional inputs without translational equivariance, the 1D-CNN leverages temporal inductive bias through localized convolutional kernels to capture continuous morphological waveforms. This architectural advantage allows the network to automatically extract physiologically relevant features rather than relying on hand-crafted engineering inputs. While a full interpretability analysis (e.g., via Gradient-weighted Class Activation Mapping, Grad-CAM) is beyond the scope of this initial feasibility study, the superior performance of the temporally aware CNN underscores the importance of preserving the sequential integrity of neuromuscular and kinematic data. The core architectural and performance metrics are summarized in Table 3; the class-wise CNN predictions are presented in Figure 7.

**Figure 8:**
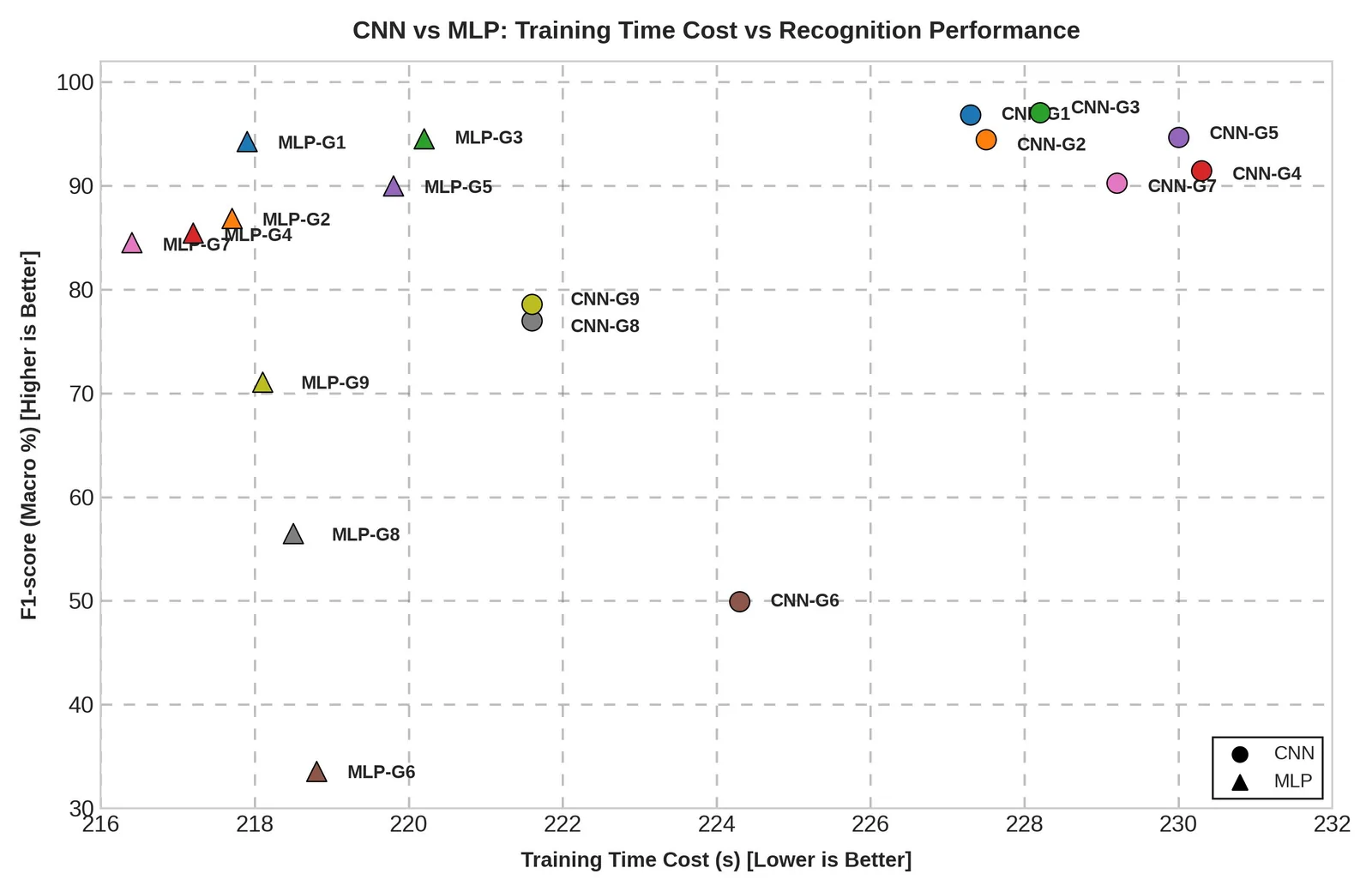
Recognition performance and training-time trade-off across the nine model and sensor configurations, measured on the A100-based environment described in Section 2.5. Despite its substantially smaller parameter count, the CNN consistently required slightly longer training time than the MLP across all nine configurations.

We repeated the complete 9-configuration ablation benchmark across 10 independent random seeds (seeds: 42, 123, 456, 789, 1011, 1337, 2024, 2026, 3141, 5555). All training and inference were conducted on the A100-based environment specified in Section 2.5. The corresponding numerical values are reported in Table 4. The recognition-performance and training-time trade-off across the nine model and sensor configurations is visualized in Figure 8.

Across the nine sensor configurations evaluated over 10 randomized seeds, the IMU+FSR configuration (G3) achieved a macro F1-score of 97.07% ±0.14% for the CNN, while the complete multimodal configuration (G1) yielded 96.82% ±0.12%. A paired t-test was conducted on the paired macro F1-scores across the 10 randomized seeds. The IMU + FSR configuration (G3) demonstrated a statistically significant but marginal numerical improvement over the complete configuration (G1) (mean difference = 0.25 percentage points; $t\left(9\right)=3.82$; p = 0.004). To verify this conclusion, the complete distribution of the 10 paired observations, the test statistic, and the degrees of freedom are detailed in Supplementary Table S1. Despite this minor numerical advantage for G3 in discrete healthy gesture classification, the complete configuration (G1) was retained as the primary platform because the integration of sEMG, pressure, and kinematics is essential for downstream multidimensional DTW interpretation.

Notably, despite possessing approximately 8.2-fold fewer trainable parameters (213,964 vs. 1,749,548) and a lower estimated per-sample forward-pass computational cost (approximately 1.21 million vs. 3.49 million floating-point operations), the CNN consistently required slightly longer wall-clock training time than the MLP across all nine sensor configurations (mean difference ≈ 8.6 s, range 3.1–13.1 s; Table 4). We attribute this counterintuitive pattern to architectural heterogeneity rather than computational load: the CNN’s twelve sequential, heterogeneous operations (two Conv1D–BatchNormalization–MaxPooling–Dropout blocks) each require an independent CUDA kernel launch with associated batch-statistic synchronization, whereas the MLP’s homogeneous stack of dense layers maps efficiently onto a small number of large, Tensor Core-optimized general matrix multiplications. This suggests that on modern data-center accelerators such as the A100, wall-clock training time is governed jointly by kernel-level execution efficiency and computational load, rather than by parameter count or FLOPs alone.

### 3.2. Paired CNN–MLP Comparison

Of 2400 test samples, both models were correct on 2228, the CNN alone was correct on 90, the MLP alone was correct on 28, and both models were incorrect on 54. McNemar’s test with continuity correction yielded χ^2^ = 31.5339 and *p* = 1.96 × 10^−8^, indicating a statistically significant difference in the paired sample-level predictions in favor of the CNN. The paired classification outcomes and McNemar test statistics are summarized in Table 5.

Because repeated samples were contributed by each participant, this test does not model within-participant clustering and should be interpreted together with the subject-level LOSO results. To account for within-participant clustering, a subject-level paired Wilcoxon signed-rank test was conducted on the 20-fold LOSO accuracies between the 1D-CNN and MLP. The 1D-CNN significantly outperformed the baseline MLP ($W=\;3.0,\;\;p\;=\;1.5\;\times \;10^{-4}\;<\;0.001$), achieving higher classification accuracy in 19 out of the 20 individual participants.

### 3.3. Subject-Independent Performance

LOSO performance was lower and more variable than sample-wise random-split performance. The CNN achieved 81.23% ± 16.74% accuracy and a macro F1-score of 79.86% ± 17.88%, whereas the MLP achieved 72.58% ± 15.59% accuracy. The large standard deviations indicate substantial interparticipant variability in sensor placement, wrist geometry, device tightness, and movement execution.

Participant-level analysis revealed accuracy ranges from 29.83% (Participant 18) to 96.33% (Participant 15). Class-wise evaluation further highlighted that gross forearm rotations and resting classes generalized well across unseen subjects (NM: 99.4%, FP: 97.7%, FS: 96.0%). Conversely, finger-specific fine manipulation exhibited higher cross-subject degradation (O: 63.5%, HG: 73.4%, TA: 81.2%), likely due to anatomical variability in wrist circumference and tendon routing altering the sEMG envelope and FSR loading dynamics. A comprehensive reporting of the subject-independent LOSO cross-validation results, detailing the classification accuracy and macro F1-score for all 20 individual held-out participants across all 12 gesture classes for both the 1D-CNN and MLP models, is provided in the Supplementary Materials (Supplementary Tables S4–S8).

To comprehensively evaluate the discriminative capability of the proposed 1D-CNN, we expanded the benchmark to include both traditional machine learning classifiers (Support Vector Machine, Random Forest) and sequence-based deep learning architectures (Bidirectional LSTM, Bi-LSTM). As shown in Table 6, all models were evaluated using the complete multimodal input configuration under identical random-split and subject-independent (LOSO) partitioning to ensure a strict comparison.

While the Bi-LSTM and Random Forest classifiers demonstrated competitive within-dataset performance, the 1D-CNN consistently outperformed all baselines. Specifically, the 1D-CNN achieved the highest random-split macro F1-score (96.59%) and the highest subject-independent LOSO accuracy (79.78% ±18.86%). This confirms that the localized temporal inductive bias leveraged by the 1D-CNN’s convolutional filters is highly effective at capturing the continuous morphological nuances of multimodal biosignals, yielding superior cross-subject generalization compared to flattened representations (MLP, SVM, RF) and recurrent architectures (Bi-LSTM). The detailed performance comparisons across all evaluated models are summarized in Table 6.

### 3.4. Re-Donning Performance

The same-participant re-donning test produced an overall accuracy of 95.8%. Eight classes achieved 100% accuracy. Hook-like grasp (82%), thumb adduction (88%), opposition (90%), and mass flexion (90%) were more sensitive to re-donning and execution variability.

**Figure 9:**
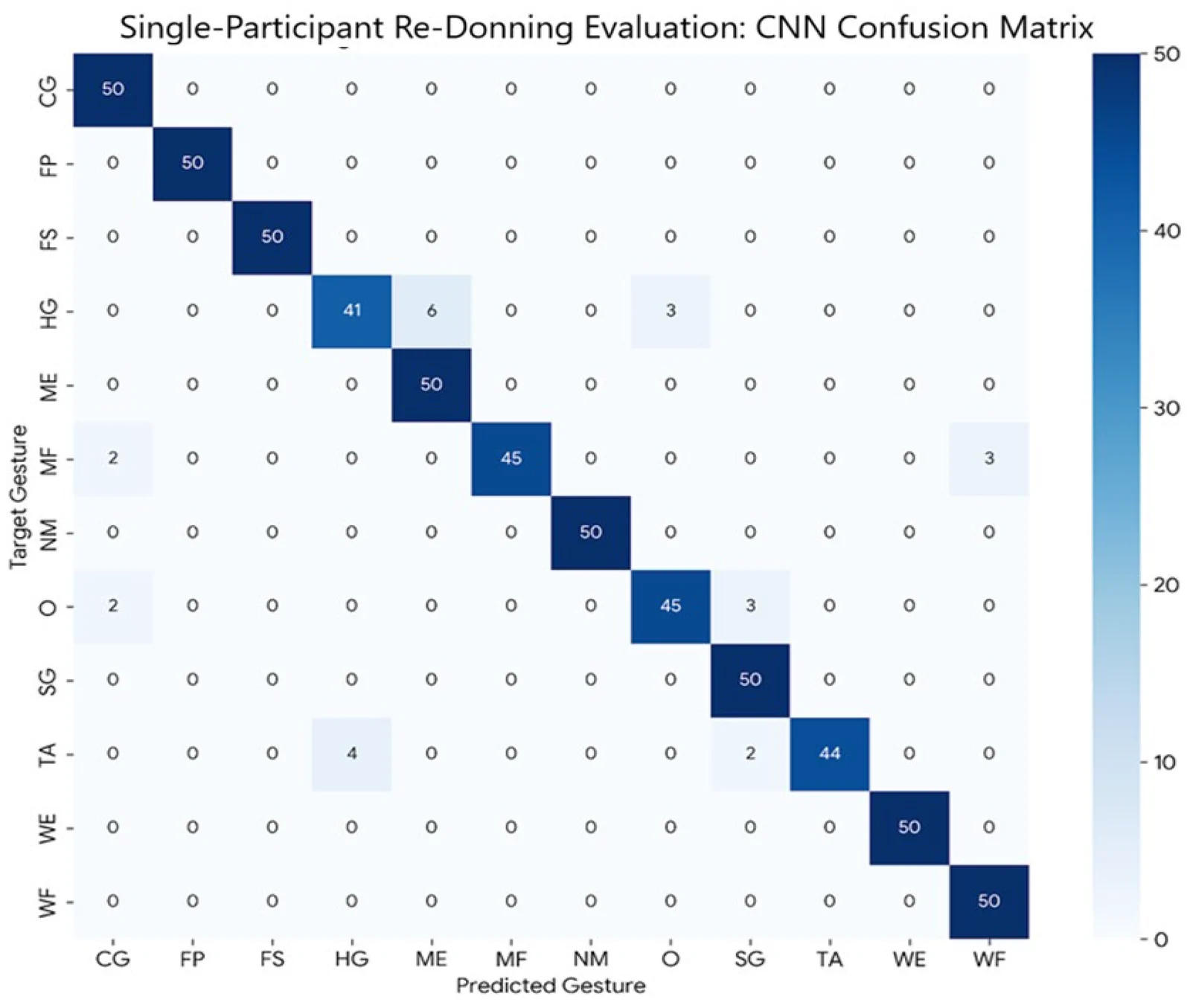
Confusion matrix of the 600-trial same-participant re-donning test.

**Figure 10:**
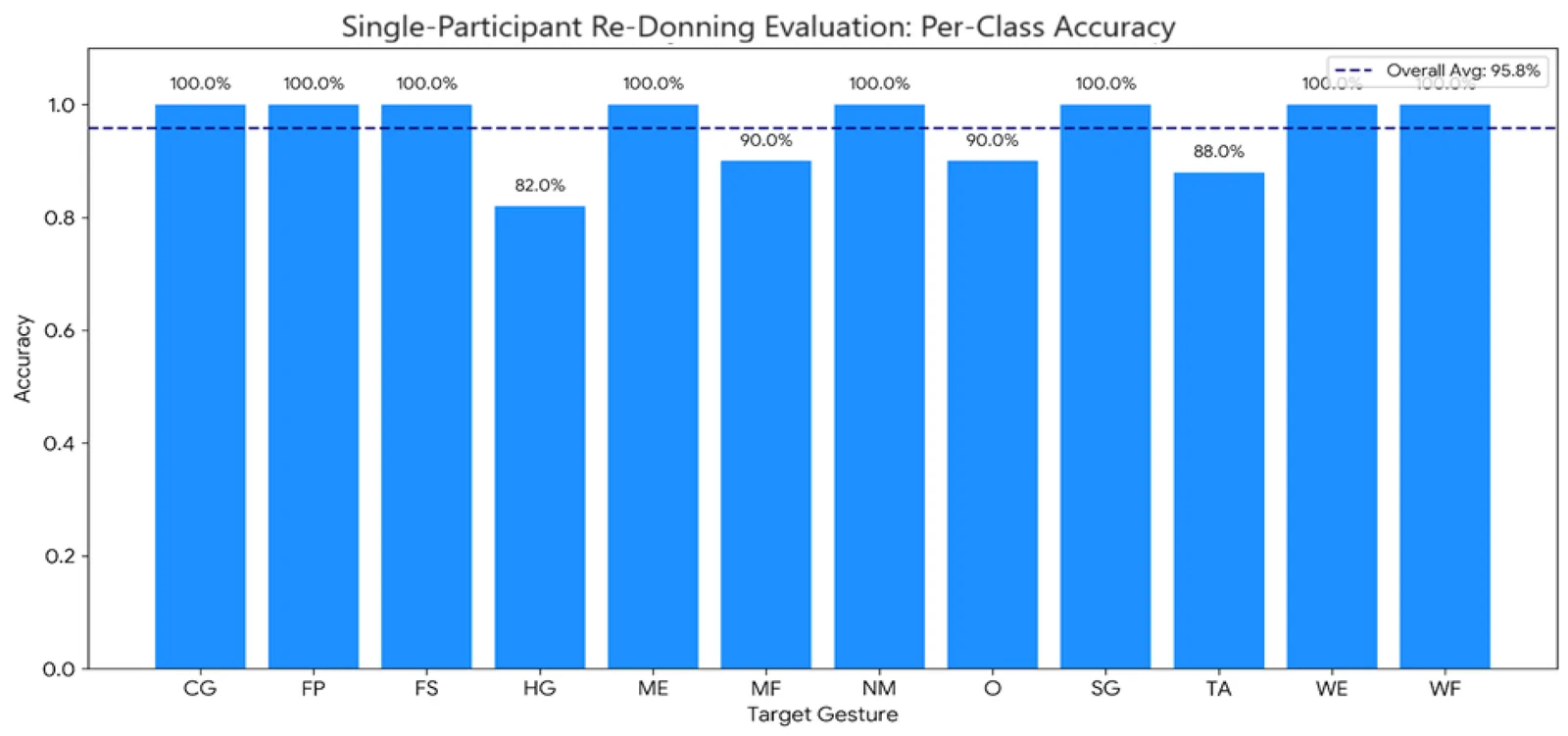
Per-class accuracy in the 600-trial same-participant re-donning test.

The re-donning confusion matrix is shown in Figure 9. Most classes remained highly stable after re-donning, whereas hook-like grasp, thumb adduction, opposition, and mass flexion accounted for most of the residual misclassifications. The corresponding per-class accuracy values are presented in Figure 10.

### 3.5. DTW Quantification and Visualization

It is important to note that within the scope of this proof-of-concept study, the DTW module is strictly proposed as a mathematical formulation and a graphical visualization framework. It is designed to highlight potential signal deviations across multiple modalities, rather than serving as a validated diagnostic classifier. DTW alignment reduced temporal mismatch between the reference and evaluated sequences and enabled synchronized inspection of the aligned waveforms and corresponding video frames. The analysis yielded a normalized DTW distance, channel-contribution percentages, and a maximal-difference segment.

**Figure 11:**
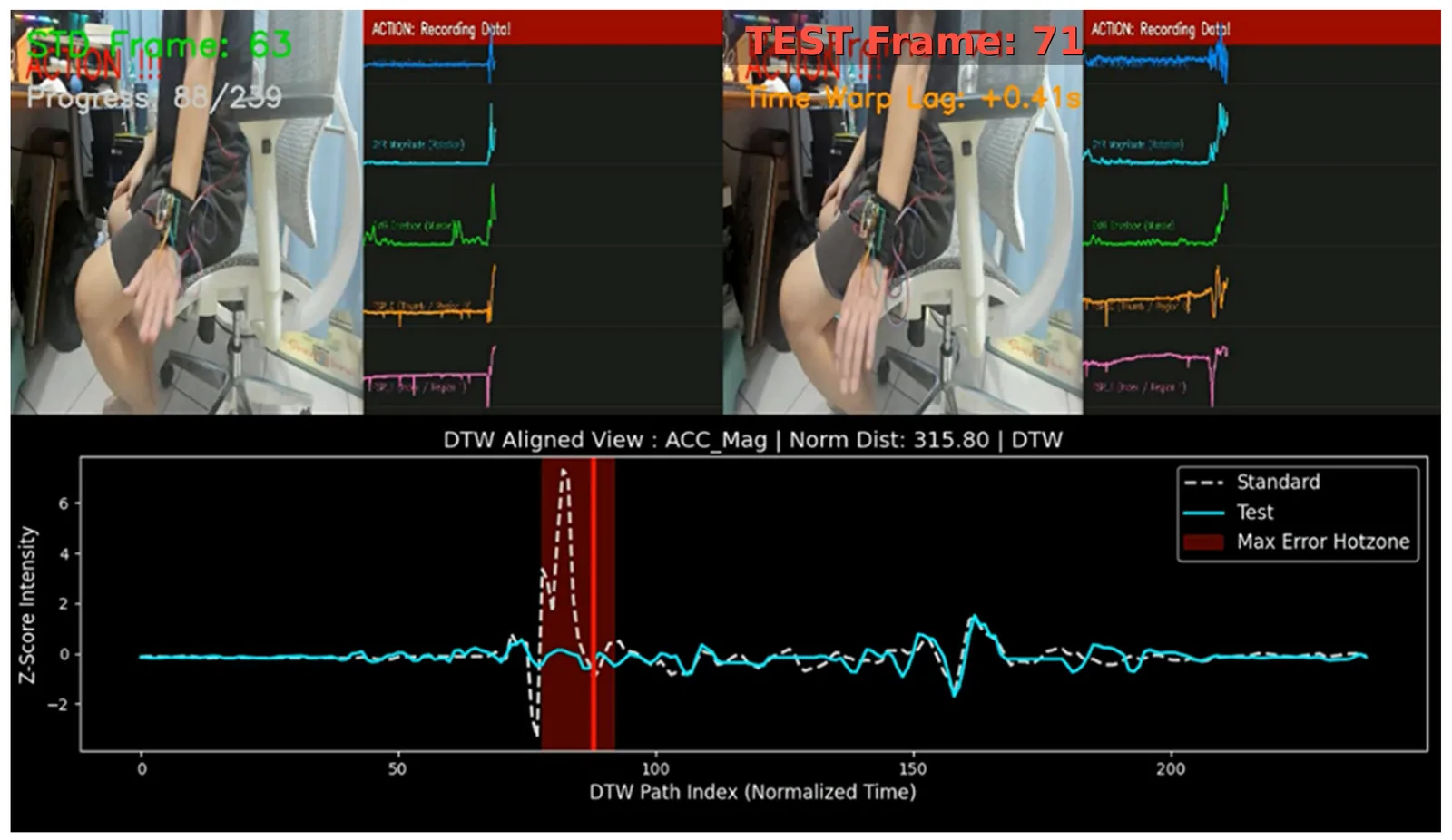
Example of DTW-aligned multimodal waveforms and synchronized video frames. Note: The interface label displaying ‘Norm Dist: 315.80′ reflects the cumulative distance prior to path-length division; the resulting normalized metric is 1.32 (315.80/239). This extended trial is presented strictly as an illustrative visualization of the GUI alignment pipeline rather than a direct quantitative benchmark against Table 7.

An example of the DTW-aligned multimodal waveforms and synchronized video frames is shown in Figure 11. It should be noted that while the standard dataset classification and the reference distributions (Table 7) strictly utilize a fixed 100-sample (1-s) window, the qualitative visualization interface intentionally loads slightly extended sequences from the continuous raw CSV buffer (e.g., ~120–150 samples). This extension visually captures the pre-movement resting baseline and post-movement relaxation phases alongside the active gesture, naturally resulting in a warping path length (e.g., L = 239) that exceeds the 199-position mathematical limit of a strict 100 × 100 alignment. To establish comparability between these extended visualization sequences and the strict 100-point reference templates, the system applies path-length normalization. While the interface label in Figure 11 indicates a distance of 315.80 corresponding to the un-normalized cumulative sum, deriving the path-length normalized metric (cumulative: 315.80, normalized: 1.32) provides an illustrative qualitative normalization of the displayed trial. Because the visualization interface intentionally incorporates surrounding resting baselines, this normalized distance is presented strictly as a visual interface demonstration rather than a direct mathematical benchmark against the 100-point reference distributions in Table 7.

Finally, when evaluating the simulated altered movements within the visualization framework, the newly introduced decoupled metrics successfully captured the execution deviations. For example, in simulated bradykinetic and hypometric trials, the Kinematic Duration Ratio ($T_{ratio}$) appropriately exceeded 1.0 (e.g., $T_{ratio}\approx 1.45$), while the Peak Amplitude Modulation ($Amp_{ratio}$) fell below 1.0 (e.g., $Amp_{ratio}\approx 0.62$), effectively quantifying the prolonged execution time and reduced physical effort that might otherwise be obscured by DTW time-warping alone.

To independently validate the robustness of these reference distributions, a nested cross-validation framework evaluated the randomly held-out test cohorts across 20 iterations. The resulting gesture-specific normative distributions and independent pass rates are summarized in Table 7.

The overall 95.47% pass rate represents the grand mean of holdout evaluations across the 20 random iterations (5 held-out subjects × 50 trials × 12 gestures per iteration). As detailed in Table 7 and the comprehensive participant-level matrix (Supplementary Tables S2 and S3), the overall independent evaluation achieved a 95.47% pass rate across all 12 gesture categories, successfully falling below their respective prospective μ+ 2σ thresholds. This remarkably high validation rate confirms the stability of the normative distributions for independent single-trial assessment. The remaining 4.53% of trials that fell above this threshold highlight the natural inter-subject anatomical and execution variability; for instance, certain individuals (e.g., P18) exhibited lower baseline pass rates due to unmitigated sensor impedance or anatomical variances under the zero-calibration protocol. Furthermore, while the normative distributions achieved a 95.47% independent pass rate across healthy trials, this high healthy-trial coverage validates normative distribution stability but does not demonstrate the empirical detection or classification of true pathological movement abnormalities, which requires dedicated clinical trials in patient cohorts. Because the protocol strictly utilized a zero-calibration (non-MVC) approach to simulate out-of-the-box home deployment, this minor variance correctly reflects baseline physiological heterogeneity rather than true pathological deviation, indicating that these thresholds can reliably capture major deviations while tolerating natural human variances.

When processing the simulated altered movements, the FastDTW algorithm successfully generated corresponding Hotzones on the visualization interface to highlight potential temporal and multimodal signal deviations. However, it is important to emphasize that within the scope of this proof-of-concept study, these outputs are proposed strictly as a qualitative graphical visualization framework. The system does not currently quantify the localization accuracy of these deviations against an independent clinical or kinematic reference (such as synchronized video or optical motion capture). Rigorous quantitative validation of the DTW Hotzone accuracy is reserved for future investigations.

## 4. Discussion

The principal contribution of this study is the integration of 12-class hand-gesture recognition with interpretable DTW-based motion-difference quantification in a compact wrist-worn system. The complete multimodal CNN outperformed the corresponding MLP in the paired random-test comparison while using substantially fewer trainable parameters. This result is consistent with the suitability of one-dimensional convolution for multichannel wearable time-series data [12].

The difference between random-split and LOSO performance provides critical insight into the physiological robustness of the system. The substantial decrease from a random-split accuracy of 96.58% to a LOSO accuracy of 81.23% ± 16.74% clearly highlights the challenge of inter-subject biological variability. A core physiological factor driving this performance drop is the utilization of a fixed engineering scaling factor rather than physiological normalization based on Maximum Voluntary Contraction (MVC). In clinical electromyography, MVC normalization is essential to compensate for individual differences in muscle mass, subcutaneous fat thickness, skin-electrode impedance, and sensor placement variations. By bypassing MVC to simulate a rapid, zero-calibration home deployment, the system inherently absorbs these unmitigated biological variances into the dataset. Consequently, the 1D-CNN faced significant ‘domain shifts’ when evaluated on unseen subjects. Future iterations must balance the usability benefits of zero-calibration against the biological fidelity gained through personalized baseline normalization.

While a preliminary observation in a single participant (*N* = 1) yielded 95.8% accuracy upon device reinstallation, this strictly serves as a limited proof-of-concept. It does not establish statistical cross-session reliability or population-level robustness against sensor displacement. Future validation should include multiple participants, repeated complete removal and reinstallation, multiple days, and leave-one-session-out analysis.

The sensor-configuration comparison requires a balanced clinical interpretation. For discrete gesture classification among healthy adults, kinematic (IMU) and contact forces (FSR) provide sufficient boundary separation, achieving comparable or slightly higher macro F1-scores than the complete configuration across multiple randomized seeds. We intentionally accept this minor classification trade-off because the primary utility of sEMG lies in identifying physiological effort independent of physical movement. In hemiparetic patients with severe paralysis, kinematic sensors often record zero displacement; thus, sEMG provides a complementary modality capable of detecting attempted contraction and residual neural drive. Furthermore, as qualitatively illustrated in our preliminary UI demonstration, simulated hypometric movements visually exhibit transient sEMG bursts alongside attenuated kinematic trajectories. This provides a candidate hypothesis that multimodal sensing could conceptually assist in differentiating restricted kinematic execution from absent voluntary drive (such as flaccid paresis), though confirming true neuromuscular integrity requires prospective electromyographic calibration in clinical populations. Therefore, while sEMG provides minimal incremental accuracy for discrete healthy gesture classification, it represents a proposed exploratory channel for clinical interpretability and motor intent detection in prospective neurorehabilitation studies. Prior studies have likewise reported the complementary value of combining electrophysiological and mechanical wearable sensing [4,7,8,9].

The DTW workflow extends classification by identifying when and through which sensing channel an evaluated movement differs from a reference. Gesture-specific reference distributions are preferable to a single global threshold because intrinsic variability differs among gestures. Comparable work has used wearable IMUs and DTW to monitor task-practice quality or assess upper-limb motion remotely [13,14]. However, the present healthy-adult distributions are reference distributions, not diagnostic thresholds, and their relationship to impairment severity, compensatory movement, spasticity, or functional recovery remains unknown.

### 4.1. Physiological and Biomechanical Implications of Multimodal Biosignal Alignment

Beyond black-box classification, the synchronized FastDTW pipeline bridges discrete machine-learning predictions with interpretable clinical biomechanics by highlighting hypothesized failure modes and candidate biomechanical correlates along the temporal alignment path:

• **Hypothesized Failure Modes in Neuromuscular Activation (**${\mathrm{EMG}}_{\mathrm{Env}}$**):** Highligts potential abnormalities in motor neural drive. A delayed or broadened sEMG peak within a Hotzone provides a candidate proxy for compensatory motor unit recruitment, prolonged discharge, or antagonistic co-contraction, whereas attenuated amplitude may serve as a hypothesis for recruitment deficiency. We emphasize that these interpretations represent candidate physiological mechanisms requiring independent clinical validation. The current standalone measurements cannot definitively prove anomalous neural drive, and single-channel sEMG cannot isolate single motor-unit firing rates or deep muscular synergies without high-density electrode arrays.
• **Candidate Biomechanical Correlates of Spatial Trajectories (ACC_Mag,GYR_Mag):** Illustrates kinetic deficits during transit. Flattened acceleration within an active Hotzone reflects movement slowing or restricted joint excursion. Because the IMU is wrist-worn, differentiating whether velocity loss stems from intrinsic wrist paresis or proximal shoulder/trunk compensation requires prospective full-body kinematic tracking.
• **Candidate Biomechanical Correlates of End-Effector Interaction (FSR_0_,FSR_1_):** Highlights variations in local tissue loading and grasp timing. Incongruent pressure peaks reveal altered physical coupling and delayed contact stabilization, reflecting interfacial mechanical dynamics rather than direct mechanoreceptive neurophysiology.

Synchronizing these three distinct physiological layers allows the system to systematically explore hypothesized failure modes related to anomalous neural drive (sEMG), restricted spatial kinematics (IMU), or degraded end-effector contact regulation (FSR), rather than making definitive diagnostic attributions.

### 4.2. Limitations and Future Work

This study has several limitations. First, all participants were young healthy adults with a skewed sex distribution (18 male, 2 female), and trials were restricted to the left hand. Physiological variations in subcutaneous adipose tissue, forearm muscle cross-sectional area, and wrist circumference across sexes and broader clinical cohorts may alter sEMG signal-to-noise ratios and FSR interfacial mechanical coupling. Consequently, claims regarding population-level generalizability are inherently constrained, and future validation must evaluate larger, sex-balanced cohorts across diverse age and clinical demographics. Second, while sample-wise random partitioning demonstrated high within-dataset discriminability, the LOSO cross-validation underscored persistent subject-independent generalization challenges under zero-calibration conditions. Third, the preliminary re-donning evaluation was conducted on a single subject (N=1) and serves only as an initial proof-of-concept for wearable repositioning. Fourth, the Grove EMG Detector and 100 Hz sampling rate were tailored for macroscopic activation envelope tracking rather than full-bandwidth motor unit action potential decomposition. Fifth, the FSR sensors captured relative contact loading rather than calibrated absolute force values. Sixth, segmented trial acquisition in this benchmark protocol utilized an inertial threshold $(\sum \left|a_{i}\right|\;\geq 18.0$) for active movements and a command-driven bypass for resting baseline trials. While this design guaranteed identical temporal onset alignment across all trials initiated from a standardized arm-down posture, continuous home-based monitoring without prescribed trial boundaries will require deploying the dual-threshold condition ($T_{\mathrm{atv}}$). This ensures that purely isometric contractions (such as static grasping without limb repositioning) are autonomously captured via the sEMG envelope trigger without requiring macroscopic kinematic acceleration. Seventh, while the ablation benchmark was repeated across 10 independent random seeds to ensure statistical robustness, the LOSO cross-validation models were each trained with a single deterministic seed per fold, and long-term multi-day tracking remains to be evaluated. Finally, deliberately altered test gestures were performed by healthy adults and do not represent true stroke-induced neurological pathologies.

Furthermore, a notable limitation of this study is the lack of quantitative validation for the Dynamic Time Warping (DTW) Hotzone analysis. While the proposed DTW pipeline successfully provides a multimodal visualization layer that highlights potential signal deviations, we currently lack the comprehensive dataset required to quantitatively evaluate its localization accuracy against an independent ground-truth reference. Consequently, the DTW visualization framework requires rigorous validation in future clinical trials before it can be reliably deployed for diagnostic assessment.

## 5. Conclusions

This study successfully developed a biosignal-driven, wrist-worn multimodal sensing system integrating sEMG, relative pressure (FSR), and inertial (IMU) sensors for upper-limb gesture recognition and exploratory multimodal physical interpretation. The temporally aware 1D-CNN effectively captured macroscopic neuromuscular and kinematic features, achieving a 96.59% macro F1-score on a sample-wise split. However, the corresponding LOSO cross-validation accuracy of 81.23% ± 16.74% highlighted the inherent challenges of inter-subject biological variability, particularly when prioritizing a zero-calibration (non-MVC) design for home deployment. Beyond discrete classification, the synchronized multimodal DTW pipeline provided an exploratory multimodal physical interpretation layer, providing an illustrative visualization of transient kinematic discrepancies and signal deviations. By establishing gesture-specific reference distributions in healthy adults, this proof-of-concept lays a technical foundation for wearable monitoring, while clinical efficacy and true pathological correlation remain to be prospectively validated in stroke cohorts.

## Figures and Tables

**Table 1:** Sensing channels used in the analysis.

Modality	Channels	Primary Interpretation	Model Input
sEMG	EMG (1)	Forearm muscle-activation trend	Yes
Pressure	FSR_0, FSR_1 (2)	Local contact-pressure and relative loading changes	Yes
Inertial	AccX–Z, GyrX–Z (6)	Linear movement and angular velocity	Yes
Orientation	Euler angles, quaternion	Stored for visualization/future analysis	No

**Table 2:** Participant and dataset summary.

Characteristic	Value
Participants	20 healthy adults
Age	27.2 ± 4.27 years (20–35)
Sex	18 male/2 female
Dominant hand	18 right/2 left
Recording side	Left hand for all participants
Gesture classes	12
Repetitions	50 per class per participant
Total samples	12,000
Window	1 s, 100 samples at 100 Hz

**Table 3:** Full multimodal CNN and MLP performance on the random test split.

Model	Architecture Summary	Parameters	Accuracy	Macro F1-Score
CNN	Conv1D × 2 + BN × 2 + Dense × 2	213,964	96.58%	96.59%
MLP	Dense × 6 + output	1,749,548	94.00%	94.01%

**Table 4:** Macro F1-score and training time across sensor configurations (Mean ± SD over 10 random seeds).

Config.	Input Channels	CNN Macro F1%	CNN Time (s)	MLP Macro F1%	MLP Time (s)
G1	EMG + 2 FSR + 6 IMU	96.82 ± 0.12	227.3 ± 6.6	94.27 ± 0.48	217.9 ± 5.2
G2	EMG + 6 IMU	94.47 ± 0.46	227.5 ± 5.3	86.87 ± 1.03	217.7 ± 4.2
G3	2 FSR + 6 IMU	97.07 ± 0.14	228.2 ± 9.9	94.54 ± 0.51	220.2 ± 7.4
G4	EMG + 2 FSR	91.46 ± 0.37	230.3 ± 7.5	85.45 ± 1.24	217.2 ± 4.0
G5	6 IMU	94.67 ± 0.33	230.0 ± 7.4	89.99 ± 0.38	219.8 ± 6.3
G6	EMG	49.91 ± 1.02	224.3 ± 8.1	33.58 ± 1.57	218.8 ± 5.7
G7	2 × FSR	90.28 ± 0.39	229.2 ± 6.4	84.52 ± 1.05	216.4 ± 5.8
G8	1 × FSR_0	76.99 ± 0.74	221.6 ± 12.2	56.48 ± 1.12	218.5 ± 4.6
G9	1 × FSR_1	78.60 ± 0.55	221.6 ± 7.7	71.10 ± 0.40	218.1 ± 5.1

**Table 5:** McNemar paired-classification contingency table.

Outcome	Number of Samples
CNN correct and MLP correct	2228
CNN correct and MLP incorrect	90
CNN incorrect and MLP correct	28
CNN incorrect and MLP incorrect	54
Continuity-corrected χ^2^	31.5339
*p*-value	1.96 × 10^−8^

**Table 6:** Comprehensive baseline performance comparison across random-split and LOSO evaluations.

Model	Random-Split Accuracy	Random-Split Macro F1	LOSO Accuracy (Mean ± SD)	LOSO Macro F1 (Mean ± SD)
SVM	86.96%	86.94%	72.77% ± 16.43%	70.95% ± 17.88%
MLP	94.00%	94.01%	72.58% ± 15.59%	70.75% ± 16.93%
Random Forest	95.46%	95.48%	76.64% ± 17.14%	75.07% ± 18.45%
Bi-LSTM	94.42%	94.46%	77.56% ± 15.91%	75.99% ± 17.20%
1D-CNN (Proposed)	96.58%	96.59%	81.23% ± 16.74%	79.86% ± 17.88%

**Table 7:** Gesture-specific healthy-adult normalized DTW reference distributions.

Gesture	Mean	SD	Mean + 2SD	P95	Pass Rate (%)
CG	1.8239	0.4310	2.6859	2.6478	95.04
FP	1.3006	0.5481	2.3968	2.6704	97.22
FS	1.4256	0.5145	2.4546	2.4331	94.32
HG	1.8375	0.589	3.0156	2.9936	93.52
ME	1.3256	0.3544	2.0343	2.0079	95.40
MF	1.4349	0.4689	2.3728	2.4339	90.00
NM	2.4139	0.3286	3.0710	2.9730	97.08
O	1.7639	0.5196	2.8032	2.7244	97.18
SG	1.8321	0.4975	2.8270	2.6391	97.82
TA	1.9262	0.4594	2.8450	2.7298	94.98
WE	1.5345	0.4696	2.4737	2.4309	96.76
WF	1.8202	0.4642	2.7486	2.6050	96.36

## Data Availability

The datasets presented in this article are not readily available because the data are part of an ongoing study.

## Supplementary Materials

The following supporting information can be downloaded at: https://www.mdpi.com/article/10.3390/bios16100590/s1. Table S1. Paired 1D-CNN classification performance (Macro F1-score) and statistical test results across 10 randomized training seeds; Table S2 Matrix: Held-out Results by Gesture and Participant (Pass Rate %); Table S3. Randomly Selected Held-Out Test Subjects Across 20 Cross-Validation Iterations; Table S4. Summary of subject-independent (LOSO) cross-validation performance over all 20 participants; Table S5. 1D-CNN: Complete Paricipant-Level and Class-Wise LOSO Accuracy; Table S6. 1D-CNN: Pooled Metrics for All 12 Gesture Classes; Table S7. MLP: Complete Participant-Level and Class-Wise LOSO Accuracy; Table S8. MLP: Pooled Metrics for All 12 Gesture Classes
