# Supplementary material for: A Biosignal-Driven Wearable System Integrating Surface Electromyography and Kinematics for Interpretable Upper-Limb Assessment
Source: Biosensors (Basel). 2026 Oct 21;16(10):590. doi: 10.3390/bios16100590 (PMC13702270; doi:10.3390/bios16100590)
Supplement: Supplementary file 1 [file biosensors-16-00590-s001.zip › biosensors-4567009-supplementary.pdf]

Supplementary Tables

Table S1. Paired 1D-CNN classification performance (Macro F1-score) and statistical test results across 10 randomized training seeds

Seed	G1 macro-F1 (%)	G3 macro-F1 (%)	G3–G1 (pp)
42	96.666480	97.211813	0.545334
123	96.913458	97.001218	0.087760
456	96.624575	97.090225	0.465650
789	96.796470	97.085725	0.289256
1011	96.794677	96.918418	0.123742
1337	96.835954	96.998980	0.163026
2024	96.799565	96.919085	0.119520
2026	96.838874	97.374463	0.535589
3141	96.911007	97.084042	0.173035
5555	97.042037	97.001574	-0.040463

Table S2 Matrix: Held-out Results by Gesture and Participant (Pass Rate %) * Note: Overall average pass rate across 20 randomized iterations (5 subjects each): 95.47%

Gesture	P1	P2	P3	P4	P5	P6	P7	P8	P9	P10	P11	P12	P13	P14	P15	P16	P17	P18	P19	P20	Overall
CG	97.67	99.00	98.00	100.0	100.0	99.00	99.33	94.40	100.0	100.0	100.0	100.0	98.00	97.60	98.75	100.0	100.0	34.00	65.25	100.0	95.04
FP	100.0.0	100.0	100.0	100.0	100.0	99.33	100.0	99.20	100.0	100.0	100.0	100.0	100.0	100.0	100.0	99.33	98.00	2.00	97.50	90.80	97.22
FS	88.67	100.0	100.0	100.0	100.0	100.0	98.33	100.0	100.0	100.0	99.71	100.0	85.00	95.20	98.75	100.0	81.00	37.00	67.50	100.0	94.32
HG	100.0.0	100.0	100.0	92.80	100.0	100.0	99.33	25.20	100.0	100.0	100.0	100.0	100.0	100.0	100.0	100.0	100.0	54.00	89.25	88.80	93.52
ME	100.0	100.0	98.00	100.0	100.0	90.00	100.0	100.0	100.0	100.0	100.0	100.0	98.00	99.60	99.25	100.0	88.00	46.00	68.50	100.0	95.40
MF	98.67	100.0	100.0	100.0	100.0	100.0	72.67	100.0	99.60	100.0	100.0	100.0	100.0	91.60	100.0	100.0	100.0	86.00	5.75	100.0	90.00
NM	94.67	93.00	100.0	100.0	100.0	96.00	95.00	97.60	98.20	95.71	100.0	94.67	98.00	100.0	98.00	99.33	95.00	95.00	92.75	100.0	97.08
O	99.67	100.0	99.00	100.0	100.0	98.33	100.0	100.0	100.0	100.0	100.0	99.33	100.0	98.40	100.0	100.0	99.00	38.00	83.50	100.0	97.18
SG	100.0	98.50	100.0	100.0	100.0	100.0	100.0	98.40	100.0	100.0	100.0	98.67	98.00	99.60	99.50	100.0	98.00	82.00	81.25	100.0	97.82
TA	96.67	92.00	100.0	98.00	84.00	99.00	96.67	96.40	100.0	99.71	99.14	96.67	100.0	98.80	100.0	99.67	96.00	63.00	66.25	99.60	94.98
WE	100.0	100.0	100.0	100.0	100.0	96.00	100.0	100.0	100.0	100.0	100.0	100.0	100.0	98.00	97.75	100.0	86.00	24.00	94.25	90.80	96.76
WF	100.0	100.0	100.0	100.0	100.0	98.00	58.00	100.0	100.0	100.0	99.60	98.00	97.71	100.0	93.00	95.20	98.75	99.00	100.0	100.0	96.36

Table S3. Randomly Selected Held-Out Test Subjects Across 20 Cross-Validation Iterations.

Iteration	1	2	3	4	5	6	7	8	9	10	11	12	13	14	15	16	17	18	19	20
Test Subjects	4, 6, 7, 10, 11	3, 9, 13, 16, 20	4, 6, 9, 10, 14	1, 9, 11, 15, 20	2, 7, 8, 15, 16	1, 2, 6, 19, 20	4, 7, 14, 16, 19	8, 9, 10, 15, 16	9, 10, 11, 13, 19	3, 8, 9, 11, 14	6, 8, 9, 18, 20	1, 2, 15, 16, 19	4, 7, 10, 12, 19	1, 7, 12, 14, 16	1, 2, 11, 19, 20	6, 10, 15, 18, 19	7, 9, 10, 14, 19	8, 11, 12, 15, 17	4, 5, 6, 9, 15	1, 9, 11, 15, 17

Table S4. Summary of subject-independent (LOSO) cross-validation performance over all 20 participants.

Model	Accuracy mean ± SD	Accuracy range	Macro-F1 mean ± SD	Macro-F1 range	Participants / trials
CNN	81.23 ± 16.74%	29.83–96.33%	79.86 ± 17.88%	25.44–96.33%	20 / 12,000
MLP	72.58 ± 15.59%	31.67–91.67%	70.75 ± 16.93%	27.19–91.71%	20 / 12,000

Table S5. 1D-CNN: Complete Participant-Level and Class-Wise LOSO Accuracy (1)

ID	NM	MF	ME	HG	TA	O	Overall (%)	Macro-F1 (%)
P1	48/50 (96.00%)	49/50 (98.00%)	45/50 (90.00%)	47/50 (94.00%)	50/50 (100.00%)	42/50 (84.00%)	94.83	94.88
P2	50/50 (100.00%)	48/50 (96.00%)	38/50 (76.00%)	49/50 (98.00%)	44/50 (88.00%)	47/50 (94.00%)	93.83	93.83
P3	50/50 (100.00%)	39/50 (78.00%)	48/50 (96.00%)	33/50 (66.00%)	32/50 (64.00%)	36/50 (72.00%)	77.50	78.41
P4	50/50 (100.00%)	39/50 (78.00%)	49/50 (98.00%)	6/50 (12.00%)	44/50 (88.00%)	46/50 (92.00%)	85.83	83.79
P05	50/50 (100.00%)	45/50 (90.00%)	49/50 (98.00%)	47/50 (94.00%)	43/50 (86.00%)	11/50 (22.00%)	88.33	87.01
P6	50/50 (100.00%)	44/50 (88.00%)	34/50 (68.00%)	49/50 (98.00%)	47/50 (94.00%)	46/50 (92.00%)	82.50	80.23
P7	50/50 (100.00%)	4/50 (8.00%)	48/50 (96.00%)	45/50 (90.00%)	42/50 (84.00%)	9/50 (18.00%)	59.00	54.46
P8	50/50 (100.00%)	41/50 (82.00%)	49/50 (98.00%)	4/50 (8.00%)	49/50 (98.00%)	24/50 (48.00%)	85.67	82.96
P9	50/50 (100.00%)	23/50 (46.00%)	50/50 (100.00%)	50/50 (100.00%)	48/50 (96.00%)	49/50 (98.00%)	92.50	92.08
P10	47/50 (94.00%)	50/50 (100.00%)	49/50 (98.00%)	47/50 (94.00%)	50/50 (100.00%)	44/50 (88.00%)	88.67	85.47
P11	50/50 (100.00%)	41/50 (82.00%)	50/50 (100.00%)	48/50 (96.00%)	29/50 (58.00%)	34/50 (68.00%)	88.00	87.80
P12	50/50 (100.00%)	50/50 (100.00%)	50/50 (100.00%)	42/50 (84.00%)	43/50 (86.00%)	27/50 (54.00%)	88.50	88.55
P13	50/50 (100.00%)	48/50 (96.00%)	48/50 (96.00%)	40/50 (80.00%)	50/50 (100.00%)	45/50 (90.00%)	89.83	89.37
P14	50/50 (100.00%)	20/50 (40.00%)	49/50 (98.00%)	49/50 (98.00%)	49/50 (98.00%)	11/50 (22.00%)	83.67	81.97
P15	50/50 (100.00%)	49/50 (98.00%)	50/50 (100.00%)	43/50 (86.00%)	50/50 (100.00%)	49/50 (98.00%)	96.33	96.33
P16	50/50 (100.00%)	41/50 (82.00%)	50/50 (100.00%)	42/50 (84.00%)	46/50 (92.00%)	49/50 (98.00%)	89.83	89.75
P17	50/50 (100.00%)	43/50 (86.00%)	46/50 (92.00%)	40/50 (80.00%)	41/50 (82.00%)	36/50 (72.00%)	86.17	86.28
P18	50/50 (100.00%)	1/50 (2.00%)	10/50 (20.00%)	31/50 (62.00%)	1/50 (2.00%)	0/50 (0.00%)	29.83	25.44
P19	49/50 (98.00%)	1/50 (2.00%)	46/50 (92.00%)	1/50 (2.00%)	6/50 (12.00%)	2/50 (4.00%)	44.83	41.70
P20	50/50 (100.00%)	16/50 (32.00%)	50/50 (100.00%)	21/50 (42.00%)	48/50 (96.00%)	28/50 (56.00%)	78.83	76.90

Table S5. 1D-CNN: Complete Participant-Level and Class-Wise LOSO Accuracy (2)

ID	CG	SG	WF	WE	FP	FS	Overall (%)	Macro-F1 (%)
P1	40/50 (80.00%)	50/50 (100.00%)	50/50 (100.00%)	49/50 (98.00%)	50/50 (100.00%)	49/50 (98.00%)	94.83	94.88
P2	48/50 (96.00%)	46/50 (92.00%)	44/50 (88.00%)	49/50 (98.00%)	50/50 (100.00%)	50/50 (100.00%)	93.83	93.83
P3	16/50 (32.00%)	19/50 (38.00%)	43/50 (86.00%)	49/50 (98.00%)	50/50 (100.00%)	50/50 (100.00%)	77.50	78.41
P4	49/50 (98.00%)	33/50 (66.00%)	49/50 (98.00%)	50/50 (100.00%)	50/50 (100.00%)	50/50 (100.00%)	85.83	83.79
P05	44/50 (88.00%)	48/50 (96.00%)	49/50 (98.00%)	44/50 (88.00%)	50/50 (100.00%)	50/50 (100.00%)	88.33	87.01
P6	47/50 (94.00%)	26/50 (52.00%)	6/50 (12.00%)	47/50 (94.00%)	49/50 (98.00%)	50/50 (100.00%)	82.50	80.23
P7	6/50 (12.00%)	0/50 (0.00%)	41/50 (82.00%)	14/50 (28.00%)	50/50 (100.00%)	45/50 (90.00%)	59.00	54.46
P8	49/50 (98.00%)	49/50 (98.00%)	49/50 (98.00%)	50/50 (100.00%)	50/50 (100.00%)	50/50 (100.00%)	85.67	82.96
P9	48/50 (96.00%)	38/50 (76.00%)	49/50 (98.00%)	50/50 (100.00%)	50/50 (100.00%)	50/50 (100.00%)	92.50	92.08
P10	48/50 (96.00%)	1/50 (2.00%)	46/50 (92.00%)	50/50 (100.00%)	50/50 (100.00%)	50/50 (100.00%)	88.67	85.47
P11	40/50 (80.00%)	40/50 (80.00%)	46/50 (92.00%)	50/50 (100.00%)	50/50 (100.00%)	50/50 (100.00%)	88.00	87.80
P12	34/50 (68.00%)	41/50 (82.00%)	48/50 (96.00%)	46/50 (92.00%)	50/50 (100.00%)	50/50 (100.00%)	88.50	88.55
P13	42/50 (84.00%)	21/50 (42.00%)	48/50 (96.00%)	50/50 (100.00%)	50/50 (100.00%)	47/50 (94.00%)	89.83	89.37
P14	42/50 (84.00%)	43/50 (86.00%)	45/50 (90.00%)	47/50 (94.00%)	49/50 (98.00%)	48/50 (96.00%)	83.67	81.97
P15	46/50 (92.00%)	44/50 (88.00%)	48/50 (96.00%)	50/50 (100.00%)	50/50 (100.00%)	49/50 (98.00%)	96.33	96.33
P16	50/50 (100.00%)	23/50 (46.00%)	49/50 (98.00%)	42/50 (84.00%)	49/50 (98.00%)	48/50 (96.00%)	89.83	89.75
P17	47/50 (94.00%)	39/50 (78.00%)	42/50 (84.00%)	47/50 (94.00%)	50/50 (100.00%)	36/50 (72.00%)	86.17	86.28
P18	0/50 (0.00%)	0/50 (0.00%)	0/50 (0.00%)	0/50 (0.00%)	39/50 (78.00%)	47/50 (94.00%)	29.83	25.44
P19	0/50 (0.00%)	17/50 (34.00%)	23/50 (46.00%)	38/50 (76.00%)	45/50 (90.00%)	41/50 (82.00%)	44.83	41.70
P20	50/50 (100.00%)	16/50 (32.00%)	48/50 (96.00%)	50/50 (100.00%)	46/50 (92.00%)	50/50 (100.00%)	78.83	76.90

Table S6. 1D-CNN: Pooled Metrics for All 12 Gesture Classes

Gesture	Support	Precision (%)	Recall (%)	F1 (%)
NM	1000	90.86	99.40	94.94
MF	1000	82.19	69.20	75.14
ME	1000	88.07	90.80	89.41
HG	1000	60.26	73.40	66.19
TA	1000	68.93	81.20	74.56
O	1000	69.40	63.50	66.32
CG	1000	74.75	74.60	74.67
SG	1000	66.74	59.40	62.86
WF	1000	91.65	82.30	86.72
WE	1000	92.86	87.20	89.94
FP	1000	97.50	97.70	97.60
FS	1000	96.48	96.00	96.24

Table S7. MLP: Complete Participant-Level and Class-Wise LOSO Accuracy (1)

ID	NM	MF	ME	HG	TA	O	Overall (%)	Macro-F1 (%)
P1	0/50 (0.00%)	50/50 (100.00%)	50/50 (100.00%)	47/50 (94.00%)	42/50 (84.00%)	46/50 (92.00%)	86.67	84.02
P2	9/50 (18.00%)	47/50 (94.00%)	49/50 (98.00%)	49/50 (98.00%)	37/50 (74.00%)	40/50 (80.00%)	81.67	80.58
P3	50/50 (100.00%)	47/50 (94.00%)	48/50 (96.00%)	15/50 (30.00%)	8/50 (16.00%)	3/50 (6.00%)	69.17	68.38
P4	50/50 (100.00%)	40/50 (80.00%)	50/50 (100.00%)	2/50 (4.00%)	11/50 (22.00%)	27/50 (54.00%)	75.17	72.45
P05	49/50 (98.00%)	44/50 (88.00%)	50/50 (100.00%)	19/50 (38.00%)	28/50 (56.00%)	2/50 (4.00%)	76.83	77.24
P6	50/50 (100.00%)	40/50 (80.00%)	47/50 (94.00%)	41/50 (82.00%)	45/50 (90.00%)	10/50 (20.00%)	69.33	66.71
P7	0/50 (0.00%)	0/50 (0.00%)	48/50 (96.00%)	34/50 (68.00%)	38/50 (76.00%)	0/50 (0.00%)	47.00	40.43
P8	50/50 (100.00%)	39/50 (78.00%)	48/50 (96.00%)	7/50 (14.00%)	34/50 (68.00%)	11/50 (22.00%)	78.00	76.42
P9	50/50 (100.00%)	35/50 (70.00%)	50/50 (100.00%)	49/50 (98.00%)	43/50 (86.00%)	46/50 (92.00%)	91.67	91.71
P10	48/50 (96.00%)	44/50 (88.00%)	48/50 (96.00%)	49/50 (98.00%)	48/50 (96.00%)	41/50 (82.00%)	78.17	74.79
P11	47/50 (94.00%)	25/50 (50.00%)	50/50 (100.00%)	48/50 (96.00%)	23/50 (46.00%)	9/50 (18.00%)	74.50	72.93
P12	50/50 (100.00%)	46/50 (92.00%)	50/50 (100.00%)	39/50 (78.00%)	33/50 (66.00%)	21/50 (42.00%)	82.83	82.27
P13	50/50 (100.00%)	50/50 (100.00%)	49/50 (98.00%)	34/50 (68.00%)	40/50 (80.00%)	44/50 (88.00%)	82.00	81.45
P14	50/50 (100.00%)	42/50 (84.00%)	50/50 (100.00%)	49/50 (98.00%)	42/50 (84.00%)	13/50 (26.00%)	77.83	75.91
P15	50/50 (100.00%)	43/50 (86.00%)	50/50 (100.00%)	44/50 (88.00%)	41/50 (82.00%)	50/50 (100.00%)	89.67	89.34
P16	50/50 (100.00%)	36/50 (72.00%)	50/50 (100.00%)	32/50 (64.00%)	42/50 (84.00%)	47/50 (94.00%)	83.33	83.45
P17	50/50 (100.00%)	39/50 (78.00%)	46/50 (92.00%)	33/50 (66.00%)	44/50 (88.00%)	36/50 (72.00%)	77.17	76.90
P18	50/50 (100.00%)	6/50 (12.00%)	6/50 (12.00%)	4/50 (8.00%)	2/50 (4.00%)	0/50 (0.00%)	31.67	27.19
P19	50/50 (100.00%)	2/50 (4.00%)	36/50 (72.00%)	6/50 (12.00%)	6/50 (12.00%)	20/50 (40.00%)	44.00	39.92
P20	0/50 (0.00%)	12/50 (24.00%)	47/50 (94.00%)	20/50 (40.00%)	24/50 (48.00%)	15/50 (30.00%)	55.00	52.95

Table S7. MLP: Complete Participant-Level and Class-Wise LOSO Accuracy (2)

ID	CG	SG	WF	WE	FP	FS	Overall (%)	Macro-F1 (%)
P1	47/50 (94.00%)	42/50 (84.00%)	50/50 (100.00%)	48/50 (96.00%)	50/50 (100.00%)	48/50 (96.00%)	86.67	84.02
P2	33/50 (66.00%)	33/50 (66.00%)	44/50 (88.00%)	49/50 (98.00%)	50/50 (100.00%)	50/50 (100.00%)	81.67	80.58
P3	22/50 (44.00%)	28/50 (56.00%)	48/50 (96.00%)	46/50 (92.00%)	50/50 (100.00%)	50/50 (100.00%)	69.17	68.38
P4	50/50 (100.00%)	22/50 (44.00%)	50/50 (100.00%)	49/50 (98.00%)	50/50 (100.00%)	50/50 (100.00%)	75.17	72.45
P05	30/50 (60.00%)	50/50 (100.00%)	43/50 (86.00%)	48/50 (96.00%)	48/50 (96.00%)	50/50 (100.00%)	76.83	77.24
P6	15/50 (30.00%)	2/50 (4.00%)	22/50 (44.00%)	46/50 (92.00%)	48/50 (96.00%)	50/50 (100.00%)	69.33	66.71
P7	2/50 (4.00%)	0/50 (0.00%)	46/50 (92.00%)	24/50 (48.00%)	50/50 (100.00%)	40/50 (80.00%)	47.00	40.43
P8	40/50 (80.00%)	40/50 (80.00%)	49/50 (98.00%)	50/50 (100.00%)	50/50 (100.00%)	50/50 (100.00%)	78.00	76.42
P9	42/50 (84.00%)	36/50 (72.00%)	49/50 (98.00%)	50/50 (100.00%)	50/50 (100.00%)	50/50 (100.00%)	91.67	91.71
P10	34/50 (68.00%)	4/50 (8.00%)	6/50 (12.00%)	47/50 (94.00%)	50/50 (100.00%)	50/50 (100.00%)	78.17	74.79
P11	13/50 (26.00%)	45/50 (90.00%)	40/50 (80.00%)	50/50 (100.00%)	49/50 (98.00%)	48/50 (96.00%)	74.50	72.93
P12	20/50 (40.00%)	46/50 (92.00%)	44/50 (88.00%)	48/50 (96.00%)	50/50 (100.00%)	50/50 (100.00%)	82.83	82.27
P13	20/50 (40.00%)	27/50 (54.00%)	42/50 (84.00%)	49/50 (98.00%)	50/50 (100.00%)	37/50 (74.00%)	82.00	81.45
P14	27/50 (54.00%)	10/50 (20.00%)	40/50 (80.00%)	49/50 (98.00%)	47/50 (94.00%)	48/50 (96.00%)	77.83	75.91
P15	41/50 (82.00%)	24/50 (48.00%)	47/50 (94.00%)	50/50 (100.00%)	49/50 (98.00%)	49/50 (98.00%)	89.67	89.34
P16	38/50 (76.00%)	27/50 (54.00%)	35/50 (70.00%)	44/50 (88.00%)	49/50 (98.00%)	50/50 (100.00%)	83.33	83.45
P17	23/50 (46.00%)	32/50 (64.00%)	35/50 (70.00%)	45/50 (90.00%)	38/50 (76.00%)	42/50 (84.00%)	77.17	76.90
P18	2/50 (4.00%)	49/50 (98.00%)	1/50 (2.00%)	0/50 (0.00%)	20/50 (40.00%)	50/50 (100.00%)	31.67	27.19
P19	0/50 (0.00%)	1/50 (2.00%)	21/50 (42.00%)	36/50 (72.00%)	46/50 (92.00%)	40/50 (80.00%)	44.00	39.92
P20	48/50 (96.00%)	8/50 (16.00%)	17/50 (34.00%)	49/50 (98.00%)	40/50 (80.00%)	50/50 (100.00%)	55.00	52.95

Table S8. MLP: Pooled Metrics for All 12 Gesture Classes

Gesture	Support	Precision (%)	Recall (%)	F1 (%)
NM	1000	80.78	80.30	80.54
MF	1000	77.45	68.70	72.81
ME	1000	93.99	92.20	93.08
HG	1000	51.41	62.10	56.25
TA	1000	57.94	63.10	60.41
O	1000	56.79	48.10	52.08
CG	1000	54.86	54.70	54.78
SG	1000	47.47	52.60	49.91
WF	1000	79.85	72.90	76.22
WE	1000	89.04	87.70	88.36
FP	1000	96.39	93.40	94.87
FS	1000	93.15	95.20	94.16
